# Transdiagnostic and symptom‐domain associations between mental illnesses and future self‐connectedness versus future self‐valence

**DOI:** 10.1002/jcv2.70143

**Published:** 2026-06-19

**Authors:** Yi Yang, Ingrid Obsuth, Jean‐Louis van Gelder, Denis Ribeaud, Manuel Eisner, Xinxin Zhu, Aja Louise Murray

**Affiliations:** ^1^ Department of Psychology University of Edinburgh Edinburgh UK; ^2^ Department of Psychiatry University of Oxford Oxford UK; ^3^ MRC Unit of Lifelong Health and Ageing UCL London UK; ^4^ Clinical Psychology Department University of Edinburgh Edinburgh UK; ^5^ Department of Criminology Max Planck Institute for the Study of Crime, Security and Law Freiburg Germany; ^6^ Institute of Education and Child Studies Leiden University Leiden Netherlands; ^7^ Jacobs Center for Productive Youth Development University of Zurich Zurich Switzerland; ^8^ Institute of Criminology University of Cambridge Cambridge UK; ^9^ Department of Psychiatry University of Edinburgh Edinburgh UK

**Keywords:** bifactor, future self‐connectedness, future self‐valence, future time perspective, transdiagnostic

## Abstract

**Background:**

Future time perspective dimensions have been linked to various mental illnesses. Given the tendency for mental illnesses to co‐occur, it is necessary to disentangle associations between future‐oriented constructs and shared versus symptom‐domain‐specific psychopathology.

**Methods:**

After conducting a factor analysis to establish an optimal factor structure of the psychopathology data from Wave 8 of the Zurich Project on Social Development from Childhood to Adulthood study (*N* = 1180; *M*_age_ ≈ 20), this study examined the associations between mental illness dimensions and two future time perspective components: future self‐connectedness and future self‐valence.

**Results:**

An S‐1 bifactor specification was selected for primary interpretation. Both future time perspective components were negatively associated with internalising, attention deficit hyperactivity disorder, psychosis‐like dimensions, and substance use, alongside a S‐1 aggression factor.

**Conclusion:**

These findings suggest transdiagnostic relevance of self‐connectedness and future self‐valence but should be considered preliminary given single‐item measurement and limited construct coverage. Replication with more comprehensive measures is warranted.

## Key Points

**What's known?**

- Future time perspective dimensions are associated with various mental illnesses and are targeted in psychological interventions.

**What's new?**

- Using S‐1 bifactor modelling, this study shows that future self‐connectedness and future self‐valence are associated with multiple mental illness symptom domains, supporting their transdiagnostic relevance.

**What's relevant?**

- Interventions that enhance future self‐connectedness and self‐valence may offer transdiagnostic benefits across internalising, attention deficit hyperactivity disorder, and psychosis‐like symptoms, as well as substance use.
- Tailoring future‐oriented therapeutic strategies to specific symptom domains examined in this study may enhance intervention efficacy.

## INTRODUCTION

Late adolescence and early adulthood are critical developmental periods characterised by the transition to independence and the acquisition of skills necessary to support this shift. Central to a successful transition are mental processes related to envisioning future goals and values, maintaining an optimistic outlook, and adjusting present actions to align with future aspirations and preserve self‐coherence. These future‐oriented mental processes are commonly studied under the construct of future time perspective, which refers to how individuals mentally engage with the future, including how they think about, evaluate, and relate to it (Kooij et al., 2018).

**FIGURE 1:**
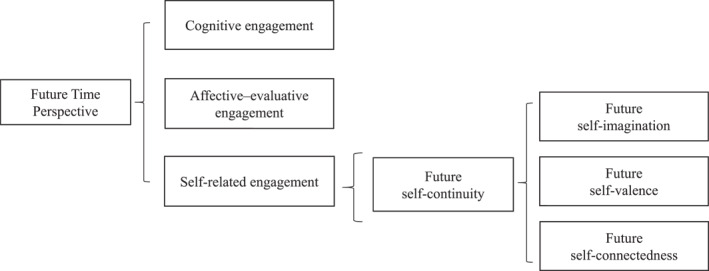
Conceptual structure of future time perspective.

Future time perspective can be conceptualised as involving multiple psychological processes, including cognitive engagement with the future (e.g., imagining future events, planning, and maintaining a future‐oriented focus), affective–evaluative engagement (e.g., forming positive or negative expectations, hope, or threat‐related appraisals), and self‐related engagement, which concerns how individuals relate to their future self, including perceived continuity or connectedness, identification with the future self, and motivation to act on behalf of that future self. As illustrated in Figure 1, future self‐continuity represents one key example of self‐related engagement within future time perspective. The diagram highlights this component as the focus of the present study and is not intended to be exhaustive, given the absence of a unified taxonomy of self‐related engagement.

Future self‐continuity refers to the subjective sense of connection between one's present and future self, reflecting a metacognitive representation of the self across time (Kooij et al., 2018; Lysaker et al., 2020; Mohammed & Marhefka, 2019; Sedikides et al., 2023). Conceptually, it can be decomposed into components including future self‐imagination vividness, future self‐valence (how positively or negatively one views the future self), and future self‐connectedness (the perceived psychological linkage between present and future selves) (e.g., Hershfield, 2011; Sokol & Serper, 2019a).

Future time perspective has been associated with a wide range of mental illnesses, generally showing small to moderate negative associations with depression and anxiety, substance use, and risky behaviour (see the meta‐analysis by Kooij et al., 2018), as well as with attention deficit hyperactivity disorder (ADHD) (Weissenberger et al., 2020), personality disorder (Mostowik et al., 2021), post‐traumatic stress disorder (PTSD, Papastamatelou et al., 2021), and compulsive behaviour (Unger et al., 2018). Future self‐continuity, a specific subdimension of future time perspective, has also been linked to mental illness. Disruptions in this dimension are commonly observed in psychiatric populations (Sokol & Serper, 2019b) and have been associated with depression, anxiety, stress, and suicidal ideation (see review by Sedikides et al., 2023), as well as psychosis (Vogel et al., 2019), schizophrenia and depersonalisation disorders (see review by Giersch et al., 2015).

One theoretical account that helps explain these associations comes from research on self‐continuity. The self has long been conceptualised as temporally extended, with continuity across past, present, and future selves forming a core aspect of self‐experience (James, 1890/1950). Self‐continuity refers to the perceived connection between one's past, present and future self and has been conceptualised as a motivational and regulatory process shaping goal‐directed behaviour and psychological functioning (Sedikides et al., 2023). From this perspective, how individuals evaluate and relate to their future self may influence behaviour, self‐control, and emotional experience, with disruptions linked to a range of mental health difficulties. Future time perspective can therefore be understood as capturing psychologically accessible aspects of how individuals evaluate and relate to their future selves; processes that are closely linked to behaviour and mental health.

Despite their theoretical and empirical support as of transdiagnostic relevance, these associations are typically reported in separate studies examining individual relationships between future time perspective constructs and individual diagnostic categories. Because symptoms frequently co‐occur and cut across diagnostic categories, findings from single‐disorder studies provide limited insight into whether associations with future time perspective generalise across comorbid symptom dimensions or are concentrated within one or a few particular symptom‐domains. Analysing disorders in isolation therefore makes it difficult to determine which symptom patterns are consistently related to future‐oriented processes and whether observed associations reflect broad cross‐diagnostic relevance or more selective links to certain symptom dimensions.

Accordingly, examining future time perspective within a framework that models multiple symptom domains simultaneously, while allowing for the possibility of a common latent structure that may capture shared variance across diagnoses or symptom domains, can clarify how these constructs relate to co‐occurring dimensions of psychopathology. By estimating both domain‐level associations and shared variance across domains within a single model, it becomes possible to evaluate whether future‐oriented processes are linked to a general liability dimension, to specific symptom domains, or to both. Such clarification has implications for interpreting prior findings and for guiding the selection of intervention targets within future time perspective dimensions.

A second gap in the literature concerns the tendency of most existing studies to examine future time perspective as a unitary construct. Disaggregating future time perspective into distinct components is clinically meaningful because different future‐oriented mental processes may map onto different psychopathological mechanisms and respond to different intervention strategies. For example, negatively biased evaluations of the future (future self‐valence) are closely linked to affective symptoms, whereas weak perceived continuity with the future self (future self‐connectedness) may contribute to impulsivity and difficulties with long‐term goal pursuit and autobiographical memory (e.g., Barry et al., 2020). Interventions may differentially influence these components, and improvement in one does not necessarily imply change in the other. Importantly, established therapies already target these distinct future‐oriented components through different therapeutic strategies, albeit not always under this label. For instance, cognitive‐behavioural therapy (CBT) primarily targets future self‐valence by modifying negatively biased expectations and catastrophic future thinking, while addressing future self‐connectedness through goal setting and future planning (Roepke & Seligman, 2016). Similarly, acceptance and commitment therapy (ACT) targets evaluative aspects of future experiences and the future self through techniques such as defusion, while strengthening continuity between present actions and future goals via values clarification and committed action (Hayes et al., 2006). Distinguishing these future‐oriented components may support more tailored and effective treatment strategies for mental illnesses with distinct underlying psychopathological mechanisms.

### Present study

Bifactor modelling approaches provide a well‐suited statistical framework for modelling general (transdiagnostic) psychopathology and specific symptom domains, and for examining their associations with candidate transdiagnostic factors such as future time perspective components (Reise, 2012). Despite growing evidence for these associations, limited research has examined how its distinct dimensions relate to multiple mental illnesses within a single transdiagnostic framework. Only one previous study (Yang et al., 2023) applied a bifactor approach but did not disaggregate specific future‐oriented dimensions (e.g., connectedness, valence), which may exhibit differential associations with mental illnesses and suggest distinct intervention targets. The objective of the present study was therefore to apply bifactor modelling to examine how two key future‐oriented components, future self‐connectedness and future self‐valence, are associated with mental illness at both transdiagnostic and symptom‐domain levels. In doing so, we aimed to provide a preliminary evaluation of how these two subdimensions relate to a range of different (co‐occurring) mental illnesses, as a first step towards understanding of the unique and shared mechanisms by which different future time perspective processes are linked to the spectrum of mental illnesses.

## METHODS

### Participants

Participants were 1180 young adults (48.1% female, 51.9% male) from the Zurich Project on Social Development from Childhood to Adulthood (z‐proso), an ongoing multi‐informant, longitudinal observational study based in Zurich, Switzerland. The study originated from a prevention and intervention initiative launched in 2004 and has since collected data across eight waves from a cohort initially recruited from 56 primary schools. Participants were enroled when they entered first grade (around age 7). Schools were selected using a stratified random sampling procedure based on school size and geographic location within Zurich.

The present study draws on Wave 8 data collected when participants were approximately 20 years old. Wave 8 was used because future self‐connectedness and future self‐valence assessment were only available at this wave, precluding longitudinal analyses involving these constructs. Most participants were born between May 1997 and April 1998, with 89.6% born in Switzerland. The cohort is socio‐economically and ethnically diverse: 58% of participants' mothers were born outside Switzerland, 53% of primary caregivers were not native German speakers, and parental education at baseline was 25.4% low, 58.4% medium, and 16.0% high. The sampling intentionally oversampled lower‐SES school districts to ensure coverage of higher‐risk neighbourhoods. The z‐proso cohort reflects the diversity of youth growing up in Zurich but is not nationally representative of Switzerland.

Ethical approval for the study was granted by the Ethics Committee of the Faculty of Arts and Social Sciences at the University of Zurich. Active informed consent was obtained from participants at age 20. Further details on recruitment procedures, sample characteristics, and study instruments are available in the cohort profile (Ribeaud et al., 2022) and the z‐proso data collection handbook (z‐proso Team, 2024). The z‐proso data and project materials can be accessed at: https://www.jacobscenter.uzh.ch/en/research/zproso/aboutus.html. Study analysis code and supplementary materials are available on the Open Science Framework: https://osf.io/8gqzh/?view_only=8fd5e53e7dab4b64bbb762cb625b08d1.

The study was preregistered on the Open Science Framework prior to accessing the data (https://osf.io/czkdq/?view_only=7c65caf950c54158bcfac5af5b17b815).

### Measures

#### Future self‐connectedness

Future self‐connectedness was assessed using a single‐item visual measure developed by Ersner‐Hershfield et al. (2009), which captures the perceived psychological linkage between the present and future self. Participants were shown seven pairs of increasingly overlapping Euler circles and asked to select the pair that best represented how connected they felt to their future self 10 years from now, with responses ranging from 1 (not connected at all) to 7 (completely connected). Prior research has shown adequate test–retest reliability over a 2‐week interval (*r* = 0.66, *p* < 0.001; *α* = 0.80) (Ersner‐Hershfield et al., 2009). The measure shows moderate convergent validity with multi‐item future self‐continuity scales (*r* ≈ 0.46–0.53) (Sokol & Serper, 2019a).

#### Future self‐valence

Future self‐valence was assessed using a single‐item pictorial measure adapted from the Self‐Assessment Manikin (Bradley & Lang, 1994). Participants were asked, ‘Which picture expresses how you feel about yourself in 10 years?’ and selected from five figures ranging from a smiling, happy figure to a frowning, unhappy figure. The SAM is a widely used pictorial measure of affective valence and arousal and demonstrates very high convergent validity with semantic differential scales (pleasure correlations ≈ 0.96–0.97; arousal ≈ 0.94–0.95), as well as clear criterion validity through correspondence with physiological and behavioural indices of affect (Bradley & Lang, 1994).

#### Social Behaviour Questionnaire

Mental illness symptoms were assessed using the Social Behaviour Questionnaire (SBQ, Tremblay et al., 1991). The scale includes 25 items assessing externalising behaviours, including symptoms of ADHD, as well as various forms of aggression (physical, indirect, instrumental/dominance, relational) and conduct problems (e.g., stealing, lying, vandalism, and oppositional/defiant behaviour). An additional 23 items from the SBQ assessed internalising problems, including symptoms of anxiety, depression, self‐harm, anger, and psychotic experiences (Murray, Eisner, & Ribeaud, 2017). Responses were provided on a 5‐point Likert‐type scale ranging from 1 (never) to 5 (very often). The SBQ has demonstrated strong psychometric properties, including reliability and validity across multiple waves of the z‐proso study (ages 7–15), and has also been validated in the current sample (Murray, Eisner, Obsuth, & Ribeaud, 2017; Murray et al., 2019).

#### Substance use

Substance use was assessed based on self‐reported frequency of use over the previous 12 months using a six‐point scale: never, once, 2–5 times, 6–12 times (‘monthly’), 13–52 times (‘weekly’), and 53–365 times (‘daily’). To ensure sufficient statistical power and avoid model convergence issues, only substances with a prevalence greater than 5% were included in the analyses. This criterion resulted in the inclusion of seven illicit psychoactive substances. Cannabinoid use was assessed with two items (cannabis/THC and cannabidiol/CBD); stimulant use with three items (ecstasy/MDMA, (meth)amphetamine, and cocaine); hallucinogen use with one item (e.g., LSD, psilocybin, and other hallucinogens); and opioid use with one item (e.g., cough syrup, pastilles, or drops containing codeine) (Quednow et al., 2022; Schifano et al., 2018).

### Statistical analysis

Because the primary aim was to examine whether future self‐valence and future self‐connectedness are associated with shared versus symptom‐domain‐specific dimensions of psychopathology, we first established an appropriate measurement structure for mental illness symptoms using a calibration–validation approach. The full sample was randomly split into two halves: the first was used for exploratory factor analysis (EFA) to identify candidate symptom domains and inform item retention, and the second for confirmatory factor analysis (CFA) to assess structural validity and generalisability (Sellbom & Tellegen, 2019; Stanton et al., 2020).

The analytic strategy therefore proceeded in five steps. First, EFA was conducted to identify a suitable latent structure of symptom domains. Second, CFA validated this structure in an independent subsample. Third, the validated symptom‐domain structure was used to specify symmetrical bifactor and S‐1 bifactor models as alternative parameterisations of shared versus symptom‐domain‐specific variance, which formed the primary analytic framework for addressing the study's transdiagnostic research questions. The optimal measurement model was selected based on model fit and interpretability. Fourth, structural equation models (SEMs) were estimated to examine associations between future self‐connectedness, future self‐valence, and symptom domains. Finally, robustness analyses assessed stability via additional SEMs for each symptom domain and within a combined oblique CFA framework.

All analyses were conducted in R (*lavaan* package; Rosseel, 2012). Given that all symptom indicators used ordered categorical response formats measuring frequency, they were treated as ordinal throughout the analyses.

#### Exploratory factor analysis

Of the 57 items across 1180 participants, only 66 values were missing (0.098%). Despite this negligible rate, multiple imputation was applied to maximise data retention and minimise potential bias, following best practices (Graham, 2009). Five imputed datasets were generated using the *mice* package (van Buuren & Groothuis‐Oudshoorn, 2011) and combined into a single dataset using the *sjmisc* package (Lüdecke, 2018). For the future self measures, the mean across imputations was computed; for ordinal variables, the modal category was selected, yielding a single representative dataset that approximates the distributional information across imputations.

To identify the latent structure of mental illness indicators (SBQ and substance use items), parallel analysis, the minimum average partial (MAP) test, and scree plot inspection were conducted. Factors were retained based on loadings ≥ |0.30|; factors with fewer than three items meeting this threshold were excluded due to limited reliability. Items with salient cross‐loadings were retained and allowed to load on multiple factors. The resulting structure informed the subsequent CFA, and items excluded at the EFA stage were not included in later CFA, bifactor, or S‐1 models to ensure analytic consistency.

#### Confirmatory factor analysis

Confirmatory factor analysis (CFA) was conducted on the validation subsample to confirm the EFA‐derived factor structure. Given that all indicators were ordinal (SBQ items: 5‐point Likert scale; substance use: six ordered categories; future self‐valence: 5‐point pictorial scale; future self‐connectedness: seven‐point ordered scale), the diagonally weighted least squares (DWLS) estimator was used (Muthén & Muthén, 2017). Missing data were minimal (<0.1%), and pairwise available observations were used to estimate the polychoric correlation matrix (missing = ‘pairwise’), consistent with *lavaan* defaults for ordinal data. Model fit was evaluated using CFI, TLI, RMSEA, and SRMR (Hu & Bentler, 1999; Pavlov et al., 2021), with acceptable fit defined as CFI and TLI >0.90 and RMSEA and SRMR <0.08.

#### Symmetrical bifactor analysis

The symmetrical bifactor model partitions symptom variance into a general factor and multiple domain‐specific group factors. All symptom items load on a single general factor that represents variance shared across all included domains, while simultaneously loading on one group factor that captures variance unique to a specific symptom domain after accounting for the general factor (Reise, 2012). This structure is widely used in psychopathology research because it separates shared transdiagnostic liability from symptom‐domain‐specific features, an important consideration given high levels of comorbidity. Longitudinal work suggests that a robust general factor is associated with earlier onset, persistence, and broader comorbidity of psychopathology (Caspi et al., 2014, 2020). In SEM applications, the general and group factors are specified as orthogonal and may be simultaneously modelled as predictors of, outcomes of, or correlated with external variables. Under this model, associations between future self‐connectedness or future self‐valence and the general factor reflect associations with statistically shared variance across symptom domains.

Based on the EFA–CFA‐validated structure, a symmetrical bifactor model was specified, with all items loading on both a general factor and one group factor, and factors constrained to be orthogonal (Gibbons & Hedeker, 1992; Reise, 2012). Model selection was guided by global fit indices and item‐level loadings (≥|0.30|). If the model did not fit well in combination with many non‐salient general factor loadings, an S‐1 bifactor specification was considered as an alternative parameterisation.

#### S‐1 bifactor analysis

Recent methodological critiques of bifactor and p‐factor approaches highlight potential estimation anomalies and interpretive limitations (Greene et al., 2023; Smith et al., 2020; Watts, 2019, 2023), motivating our use of an S‐1 specification as an alternative. Symmetrical bifactor models assume that a single general factor meaningfully accounts for shared variance across all symptom domains; however, this assumption is often not empirically supported. The extent to which a general factor emerges may depend on the specific symptom domains and measures included in the model. When the general factor is weak or unevenly supported, bifactor models may show poorer fit or estimation problems relative to correlated‐factor CFAs (Eid et al., 2017), including unstable loadings, negative variances, and interpretational difficulties (Eid et al., 2017, 2018, 2020; Heinrich et al., 2021). Such patterns indicate limited support for a fully general psychopathology factor spanning all domains.

The bifactor S‐1 model was therefore developed within the bifactor approach for situations in which the assumption of a fully general factor is not empirically supported (Eid et al., 2017, 2018, 2020). Rather than requiring all domains to define a single general factor, the S‐1 approach specifies one theoretically coherent reference domain whose items define a S‐1 factor. Other domains are included in the S‐1 bifactor portion only if they share meaningful variance with this reference domain; remaining domains are modelled outside the bifactor structure.

Consequently, the ‘general’ factor in an S‐1 model differs from that in a symmetrical bifactor model: it reflects variance shared with the reference domain rather than general psychopathology across all domains. The S‐1 approach is recommended when support for a single overarching general factor is limited or uneven across symptom domains and has been increasingly adopted in psychopathology research for this reason (e.g., Eid et al., 2017, 2018, 2020; Haywood et al., 2021; Heinrich et al., 2021; Junghänel et al., 2020).

**FIGURE 2:**
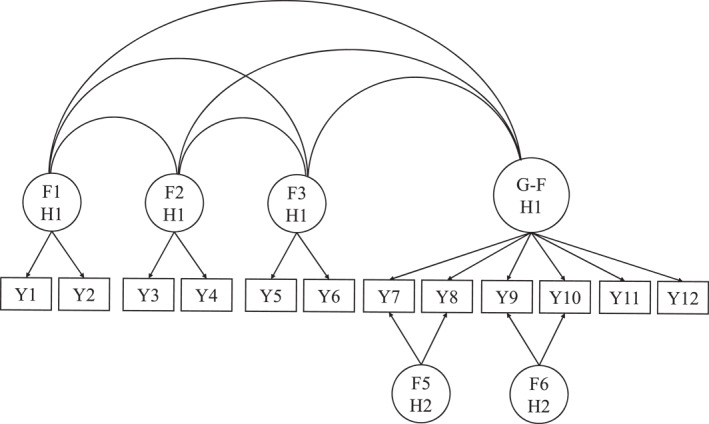
S‐1 bifactor measurement structure (schematic). Circles represent latent factors and squares represent observed indicators. G‐F (H1) denotes the partially general factor defined by the reference domain. F1–F3 (H1) represent first‐order latent factors modelled outside the bifactor portion, whereas F5 and F6 (H2) denote residual subfactors within the S‐1 bifactor structure. ‘H1’ indicates first‐order latent factors and ‘H2’ indicates residual subfactors within the bifactor portion. Single‐headed arrows represent factor loadings; curves indicate associations among latent factors. This figure illustrates the model structure and does not display parameter estimates.

Following this framework, we evaluated the S‐1 bifactor parameterisation as an alternative to the symmetrical bifactor model. The S‐1 factor, defined by a theoretically coherent reference domain, serves as the general factor within the S‐1 specification, with other symptom domains included in the bifactor portion only if they contribute meaningful shared variance and remaining domains modelled as correlated factors outside the bifactor structure. For example, an S‐1 bifactor model of psychopathology may include a bifactor structure for internalising problems (with group factors for anxiety and depression), with the general factor being correlated with other psychopathology factors such as psychosis and externalising problems. Model selection was based on global fit indices, item‐level parameter estimates, and comparison with the symmetrical bifactor specification. Figure 2 provides a schematic overview of the S‐1 bifactor portion (right) and the correlated factors outside the bifactor structure (left).

#### SEM analyses

To examine associations between future self‐connectedness, future self‐valence, and multiple mental health dimensions, we further compared the findings between SEMs using symmetrical and S‐1 bifactor measurement models. The optimal model, selected based on fit and interpretability, was used for the primary analyses, with the general (or partially general) factor and group‐specific subfactors allowed to covary with future self‐connectedness and future self‐valence.

#### Robustness analyses

To assess stability, additional SEMs were conducted. Models were estimated for each of the seven group factors individually, as well as within combined oblique CFA framework, irrespective of which of the symmetrical or S‐1 bifactor model was selected as optimal.

## RESULTS

### Exploratory factor analysis

Forty‐eight items from SBQ and seven substance use items were analysed using EFA in the calibration half of the sample (*N* = 593). Parallel analysis suggested a seven‐factor solution, whereas the MAP test indicated eight factors. Salient item loadings were defined as ≥ |0.30| (see Supporting Information S1: Table S1). Two items with loadings below this threshold and one factor (Factor 8) with only two salient items were excluded from subsequent CFAs. Four items that showed cross‐loadings were retained and assigned to both factors on which they loaded. This process resulted in a seven‐factor oblique structure comprising 51 items. Six of the extracted factors aligned closely with the theoretical SBQ subscales, based on the pattern of highest‐loading items. These factors were labelled: internalising symptoms (e.g., ‘worried’, ‘felt useless’, ‘sad without reason’, ‘felt like did everything wrong’), ADHD symptoms (e.g., ‘easily distracted’, ‘restless’), physical aggression (e.g., ‘hit, bite, kick others’), reactive aggression (e.g., ‘aggressive when teased’), indirect and proactive aggression (e.g., ‘bad things behind back’, ‘boss others around’), and psychosis‐like symptoms (e.g., ‘heard voices when alone’, ‘felt persecuted’). The remaining factor was labelled substance use, as it consisted of all seven substance use items. Detailed item contents for the seven factors are provided in Supporting Information S1: Table S2.

Supporting Information S1: Table S3 presents Pearson correlations among mental illness factors in the validation subsample (*N* = 587). Factors were defined by the item groupings identified in the EFA conducted in the calibration subsample (*N* = 593).

### Confirmatory factor analysis

The seven‐factor oblique structure identified through EFA was cross‐validated using CFA on the validation half of the sample (*N* = 587). Items were specified to load on the same factors as in the EFA, and inter‐factor correlations were freely estimated, consistent with the assumption of correlated latent constructs. The CFA model demonstrated good fit to the data (see Supporting Information S1: Table S4), and item loadings for all seven factors were generally salient (≥|0.30|), with one reactive aggression item showing a small negative loading (Supporting Information S1: Table S5).

### Bifactor analysis

Symmetrical bifactor modelling was conducted by extending the previously validated CFA model to include a model‐defined general factor representing shared variance across symptom domains. Several global fit indices met conventional thresholds (CFI = 0.912, TLI = 0.904, RMSEA = 0.052), while the SRMR was comparatively high (SRMR = 0.096; see Supporting Information S1: Table S4). Examination of item‐level parameters indicated that 18 items did not meet the conventional loading threshold of |0.30| on the general factor and/or their respective group factor, and that two group‐factor loadings were non‐significant, suggesting that it may not represent a fully general psychopathology (p‐) factor. Although the fit indices indicated that the symmetrical bifactor model provided an acceptable representation of the data with a general factor, item‐level diagnostics suggested interpretability limitations relative to alternative specifications. This pattern may reflect the specific symptom domains and measures included in the present study, as the emergence of a general factor can depend on the composition of the measurement model.

On this basis, bifactor S‐1 models were further explored to obtain a more interpretable latent structure, as the symmetrical bifactor model was not wholly empirically supported (Eid et al., 2017, 2018, 2020). The final S‐1 bifactor comprised a five‐factor structure. In this specification, physical aggression was retained as the reference domain defining an S‐1 aggression factor, while reactive aggression and indirect/proactive aggression were specified as residual subfactors within the S‐1 bifactor portion of the model. This being the optimal structure to emerge, is consistent with the predominance of aggression‐related items within the SBQ. The remaining symptom domains (internalising, ADHD, and psychosis‐like symptoms, and substance use) were modelled as correlated latent factors outside the bifactor structure.

This configuration supports a coherent interpretation of the S‐1 aggression factor, as all components of the S‐1 bifactor portion represent subtypes of aggression; accordingly, it was labelled the aggression factor and should not be interpreted as a fully general psychopathology (p‐) factor. Most items loaded positively and significantly on the aggression factor and their respective subfactors, exceeding |0.30|. Two items fell slightly below this threshold but remained statistically significant. One reactive aggression item loaded negatively on the S‐1 aggression factor, and one internalising item showed a small, non‐significant loading, both consistent with the multifactor oblique CFA results. Model fit indices indicated good fit (CFI = 0.956, TLI = 0.952, RMSEA = 0.035, SRMR = 0.069; see Supporting Information S1: Table S4), with the acceptable SRMR indicating small residuals between observed and model‐implied correlations. This S‐1 bifactor model was therefore adopted as the optimal measurement framework and used to estimate associations with future self‐valence and connectedness in the main analyses.

### SEM models

Having established an appropriate latent structure for psychopathology, we focus below on the associations between future self‐connectedness, future self‐valence, and specific symptom domains.

#### Symmetry bifactor SEM

The p‐factor was significantly associated with both future self‐valence (*r*_p‐factor_ = −0.358, *p* < 0.001, [95% CI = −0.436, −0.279]) and future self‐connectedness (*r*_p‐factor_ = −0.173, *p* < 0.001, [95% CI = −0.263, −0.083]). Again, this model was not selected for primary interpretation, as item‐level diagnostics indicated that it provided a less interpretable representation of the data relative to alternative specifications (Supporting Information S1: Table S6; Figure S1).

#### S‐1 bifactor SEM

**FIGURE 3:**
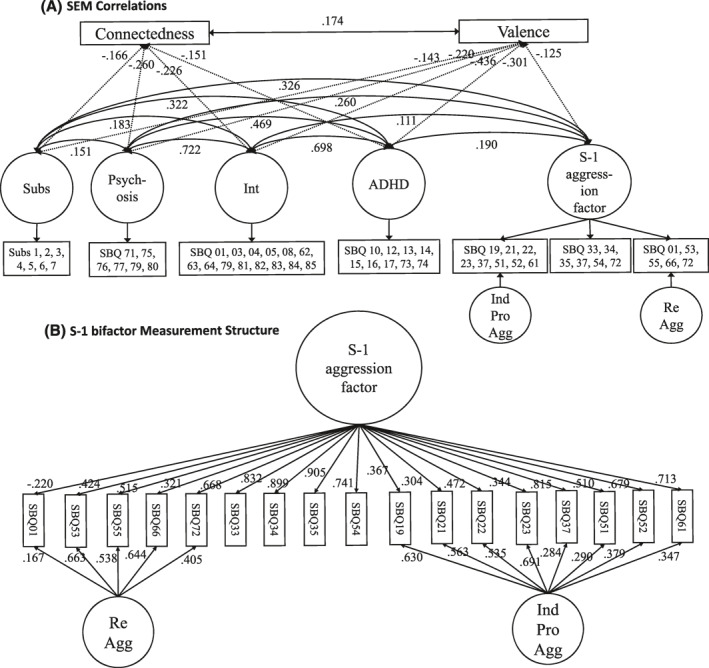
S‐1 bifactor measurement and structural model of psychopathology. IndProAgg, indirect and proactive aggression; Int, internalising symptoms; PhyAgg, physical aggression; ReAgg = reactive aggression; Subs, substance use. (A) Circles represent latent symptom subfactors. Solid arrows indicate positive structural associations (standardised estimates), and dashed arrows indicate negative associations. All connections between latent factors represent correlations (bidirectional paths), not directional loadings. The dashed boundary highlights the S‐1 bifactor portion of the model, in which physical aggression serves as the reference domain defining a S‐1 aggression factor. This factor reflects shared variance within aggression‐related domains. Additional covariances are also observed: between IndProAgg and Int, attention deficit hyperactivity disorder (ADHD), and Psychosis; between ReAgg and Int, ADHD, and Psychosis (see Supporting Information S1: Table S7). All parameters were significant at *p* < 0.001 except: connectedness–psychosis, connectedness–substance use, valence–substance use (*p* < 0.01); valence–S‐1 aggression factor, internalising–S‐1 aggression factor, psychosis–substance use (*p* < 0.05). (B) The S‐1 bifactor measurement structure showing the partially general aggression factor and residual aggression subfactors. Rectangles represent observed indicators, and solid arrows represent factor loadings. All item loadings are significant at *p* < 0.001.

Model fit was good across all indices, as shown in Supporting Information S1: Table S4. Item loadings and factor associations for the S‐1 models are presented in Supporting Information S1: Table S7 and Figure 3. Future self‐valence was negatively associated with the S‐1 aggression factor (*r*_aggression_ = −0.125, *p* < 0.05, [95% CI = −0.223, −0.028]) and all 4 non‐aggression factors (*r*_internalising_ = −0.436, *p* < 0.001, [95% CI = −0.510, −0.362]; *r*_ADHD_ = −0.301, *p* < 0.001, [95% CI = −0.380, −0.222]; *r*_psychosis_ = −0.220, *p* < 0.001, [95% CI = −0.327, −0.113]; *r*_substance_ = −0.143, *p* < 0.01, [95% CI = −0.246, −0.041]). In addition, future self‐connectedness was negatively associated with all 4 non‐aggression factors (*r*_internalising_ = −0.226, *p* < 0.001, [95% CI = −0.307, −0.145]; *r*_ADHD_ = −0.151, *p* < 0.001, [95% CI = −0.231, −0.070]; *r*_psychosis_ = −0.260, *p* < 0.001, [95% CI = −0.359, −0.161]; *r* _substance_ = −0.166, *p* < 0.01, [95% CI = −0.265, −0.067]), but not with the S‐1 aggression factor (*r*_aggression_ = −0.085, *p* = 0.084, [95% CI = −0.181, 0.011]). Further, future self‐connectedness and future self‐valence were positively correlated (*r* = 0.174, *p* < 0.001, [95% CI = 0.096, 0.252]).

### Robustness analyses

As shown in Supporting Information S1: Table S8 and Figure S2, the SEM results based on the multifactor oblique CFA model showed a broadly similar pattern of symptom‐domain‐specific associations between future self‐connectedness, future self‐valence, and specific symptom domains to those observed in the S‐1 bifactor SEM (see Supporting Information S1: Appendix S1 for details). Separate SEMs conducted for each symptom domain (Supporting Information S1: Tables S9–S15) confirmed these associations. Across models, future self‐valence and future self‐connectedness were negatively associated with all symptom domains except physical aggression.

The study was preregistered prior to data access and specified the use of bifactor modelling to examine shared versus symptom‐domain‐specific variance, with model evaluation guided by fit and interpretability. Exploratory and confirmatory factor analyses were conducted to establish an appropriate measurement structure prior to bifactor modelling. The S‐1 bifactor specification was not explicitly named in the preregistration but was evaluated within the broader preregistered bifactor framework and selected based on prespecified model comparison criteria. Further details are provided in Supporting Information S1: Table S16.

## DISCUSSION

The present study examined associations between future self‐valence, future self‐connectedness, and mental illness while accounting for covariation among symptom domains. To investigate both transdiagnostic and symptom‐domain‐specific associations, we employed factor modelling approaches that aim to separate domain‐general and domain‐specific variation. The S‐1 bifactor SEM revealed that both future self‐valence and future self‐connectedness were negatively associated with internalising symptoms, ADHD symptoms, psychosis‐like symptoms, and substance use, with additional associations observed for certain forms of aggression. These findings are consistent with the potential transdiagnostic relevance of future‐oriented self‐concepts.

One way to interpret these findings is through the lens of self‐continuity, which conceptualises connections to the future self as central to motivation, self‐regulation, and emotional functioning. From this perspective, future self‐valence reflects how individuals evaluate their future, with more negative expectations linked to hopelessness, anxiety, and reduced anticipation of positive outcomes. Such evaluative biases are central to internalising symptoms, where individuals may anticipate negative outcomes, discount positive future possibilities, and engage in maladaptive worry or rumination. In contrast, future self‐connectedness reflects the perceived continuity between present and future selves, which supports goal‐directed behaviour and self‐regulation. Weaker connectedness may reduce the salience of future consequences, contributing to impulsivity and preference for immediate rewards, processes implicated in ADHD symptoms and substance use. More broadly, disruptions in future self‐continuity may also affect the integration of temporal self‐experience, potentially contributing to difficulties in maintaining coherent expectations about the future, which have been observed in psychosis‐like symptoms. Disruptions in these processes may therefore contribute to maladaptive patterns across the multiple symptom domains.

Both future self‐valence and future self‐connectedness were negatively associated with internalising symptoms, in line with previous evidence linking future‐oriented cognition to affective outcomes (Kooij et al., 2018; Sokol & Serper, 2017; Wang et al., 2022). Consistent with prior research (see review by Sedikides et al., 2023), these future self constructs were also associated with psychosis‐like symptoms. Previous studies have shown that schizophrenia and psychosis are linked to reduced positive and negative future‐oriented thinking (e.g., Barry et al., 2020; Goodby & MacLeod, 2016), and that deficits in future self‐connectedness relate to symptom severity and functional impairment (Sedikides et al., 2023). These disruptions have been proposed as central to psychosis, potentially reflecting impairments in temporal integration and the synchronisation of inner time with external and social processes (Fuchs, 2013; Giersch et al., 2015; Mitchell et al., 2021; Thoenes & Oberfeld, 2017; Vogel et al., 2019; Vogeley & Kupke, 2007). Such impairments may contribute to hallucinations and delusions through attempts to restore perceptual coherence (Postmes et al., 2014).

The present study also contributes to the accumulating evidence on the association between negative future self‐valence, future self‐connectedness, and substance use. Previous studies have mainly focused on common substances (e.g., alcohol, tobacco) or a mix of common and novel substances (e.g., Kooij et al., 2018). These associations may be explained in terms of repetitive reward and reinforcement processes, which are core features of dependency and addiction (e.g., DSM‐5; ICD‐11). Substance use may provide more immediate reinforcement by facilitating access to hedonic experiences or by enabling escape from negative affect.

Despite the large volume of studies on time perception and estimation in ADHD (Mette, 2023; Nejati & Yazdani, 2020; Ptacek et al., 2019), to the best of our knowledge only a few studies have examined the association between ADHD symptoms and future time perspective (e.g., Carelli & Wiberg, 2012; Settanni et al., 2018; Weissenberger et al., 2016, 2020; Yang et al., 2023). No existing studies have examined the relationship between ADHD and future self‐connectedness or self‐continuity. Our findings therefore addressed this gap, showing a positive association between ADHD symptoms and future‐self disconnectedness. This is important because differences in future‐oriented mental processes may be related to impaired time perception (e.g., time estimation, discrimination, management and reproduction) in association with ADHD symptoms (see reviews by Mette, 2023; Nejati & Yazdani, 2020; Ptacek et al., 2019), although further research is needed to clarify these mechanisms.

Future time perspective dimensions are important to investigate in relation to mental illness because of their intervention potential. Crucially, previous research has suggested that these dimensions are malleable. In addition to the aforementioned strategies from CBT and acceptance ACT (Hayes et al., 2006; Roepke & Seligman, 2016), training in episodic future thinking has been shown to be an effective approach for improving future‐oriented mental processes (Rung & Madden, 2018; Schacter et al., 2017; Scholten et al., 2019; Ye et al., 2022). Cognitive bias modification techniques (e.g., using the dot‐probe paradigm, ambiguous scenarios paradigm, word sentence association paradigm) may also redirect attention towards positive future‐oriented cues (e.g., words and pictures) rather than neutral or negative information that is more automatically processed (Jones & Sharpe, 2017). As proposed by Roepke and Seligman (2016), future‐oriented strategies, such as goal‐setting and planning approaches used in CBT, hold strong potential as transdiagnostic interventions across mental illnesses.

A symmetrical bifactor model demonstrated acceptable global fit, and both future self‐valence and future self‐connectedness were negatively associated with the general factor under that specification. However, item‐level diagnostics suggested that an S‐1 bifactor specification was a better representation of the data. This is consistent with the fact that, whether a general factor emerges, and how strongly it is represented, depends on the symptom domains and measures included in the model. In the present study, the SBQ provides uneven coverage across domains, with aggression relatively over‐represented. The S‐1 bifactor model was therefore selected as the optimal measurement model in which to explore associations with future time perspective. This model included an S1‐aggression factor with two aggression group factors and correlated internalising, ADHD, psychosis‐like and substance use factors. It is worth emphasising that bifactor and S‐1 models are treated as statistical parameterisations for partitioning shared and domain‐specific variance, rather than as direct tests of etiological theories of psychopathology. Although the specific S‐1 bifactor specification and S‐1 aggression factor were not preregistered, its emergence is consistent with preregistered analytic aims and with prior evidence that externalising symptoms often contribute disproportionately to shared variance in youth psychopathology (Murray, Eisner, Obsuth, & Ribeaud, 2017; Murray et al., 2019).

### Limitations and future directions

Several limitations of the present study should be noted. First, mental illness symptoms were assessed using measures selected for a general community sample and do not constitute clinical diagnostic tools. Second, future self‐connectedness and future self‐valence were each assessed using single‐item measures due to questionnaire‐length constraints in this large‐scale longitudinal cohort study. Although these items have demonstrated validity in prior research, the future self‐connectedness measure provides relatively coarse indicator of the construct. Future studies would benefit from using multi‐item instruments to capture these dimensions with greater precision and to examine additional facets of future‐oriented processes. Additionally, for symptom domains with more limited prior research on future‐oriented processes (e.g., ADHD), the observed associations should be interpreted cautiously, as it is more difficult to contextualise findings within the existing literature. Third, reference periods varied across measures, despite all data being collected within the same wave. For example, ADHD symptoms were reported with respect to the past 12 months, internalising symptoms with respect to the past month, and future self‐connectedness and self‐valence items were framed in terms of current attitudes or perceptions (Kooij et al., 2018; Mohammed & Marhefka, 2019; Shipp et al., 2009). Fourth, the present study used cross‐sectional data; therefore, only correlational associations could be examined. To further establish the intervention potential of improving future‐oriented mental processes to ameliorate mental illness, future research could employ randomised controlled trials or longitudinal designs with multiple waves of data to assess prospective associations between future‐oriented processes and mental health outcomes.

## CONCLUSION

Our findings suggest that future self‐connectedness and future self‐valence are associated with a range of mental illness symptom domains, illustrating their a transdiagnostic relevance. Incorporating disorder‐tailored intervention strategies to improve future‐oriented mental processes may enhance the prevention and treatment of different mental illnesses.

## AUTHOR CONTRIBUTIONS

**Yi Yang**: Conceptualization; methodology; visualization; formal analysis; writing—original draft. **Ingrid Obsuth**: Writing—review and editing; supervision. **Jean‐Louis van Gelder**: Methodology; writing—review and editing. **Denis Ribeaud**: Project administration; resources; writing—review and editing. **Manuel Eisner**: Methodology; writing—review and editing; resources. **Xinxin Zhu**: Writing—review and editing; methodology. **Aja Louise Murray**: Methodology; writing—review and editing; supervision.

## CONFLICT OF INTEREST STATEMENT

The authors declare no conflicts of interest.

## ETHICAL CONSIDERATIONS

Ethical approval for the z‐proso longitudinal study was granted on 12 March 2018 by the Ethics Committee of the Faculty of Arts and Social Sciences, University of Zurich (Reference No. 2018.2.12). Active informed consent was obtained from participants at age 20.

## Supporting information

Supporting Information S1

## Data Availability

The present study used data obtained from the Zurich Project on Social Development from Childhood to Adulthood (z‐proso) study for all analyses. More information on obtaining access to z‐proso data is available at: https://www.jacobscenter.uzh.ch/en/research/zproso/aboutus.html.
