# Supplementary material for: Transdiagnostic and symptom‐domain associations between mental illnesses and future self‐connectedness versus future self‐valence
Source: JCPP Adv. 2026 Jun 19:e70143. Online ahead of print. doi: 10.1002/jcv2.70143 (PMC13339054; doi:10.1002/jcv2.70143)
Supplement: Supplementary file 1 — Supporting Information S1 [file JCV2-9999-e70143-s001.docx]

**Transdiagnostic and Symptom-Domain Associations Between Mental Illnesses and Future Self-Connectedness Versus Future Self-Valence**

**Supporting Information**

| **Table S1. EFA results (N=593)** | | | | | | | | |
| --- | --- | --- | --- | --- | --- | --- | --- | --- |
| Loadings: | MR1 | MR4 | MR5 | MR2 | MR3 | MR6 | MR8 | MR7 |
| SBQ08 | 0.807 |  |  |  |  |  |  |  |
| SBQ64 | 0.722 |  |  |  |  |  |  |  |
| SBQ05 | 0.579 |  |  |  |  |  |  |  |
| SBQ85 | 0.765 |  |  |  |  |  |  |  |
| SBQ83 | 0.713 |  |  |  |  |  |  |  |
| SBQ84 | 0.562 |  |  |  |  |  |  |  |
| SBQ03 | 0.525 |  |  |  |  |  |  |  |
| SBQ04 | 0.544 |  |  |  |  |  |  |  |
| SBQ81_imp | 0.712 |  |  |  |  |  |  |  |
| SBQ15 |  | 0.740 |  |  |  |  |  |  |
| SBQ16 |  | 0.732 |  |  |  |  |  |  |
| SBQ74 |  | 0.585 |  |  |  |  |  |  |
| SBQ12_imp |  | 0.604 |  |  |  |  |  |  |
| SBQ17_imp |  | 0.738 |  |  |  |  |  |  |
| SBQ13_imp |  | 0.619 |  |  |  |  |  |  |
| SBQ14_imp |  | 0.646 |  |  |  |  |  |  |
| SBQ21 |  |  | 0.616 |  |  |  |  |  |
| SBQ52 |  |  | 0.597 |  |  |  |  |  |
| SBQ19_imp |  |  | 0.700 |  |  |  |  |  |
| SBQ22_imp |  |  | 0.684 |  |  |  |  |  |
| SBQ23_imp |  |  | 0.658 |  |  |  |  |  |
| SBQ34 |  |  |  | 0.805 |  |  |  |  |
| SBQ35 |  |  |  | 0.764 |  |  |  |  |
| SBQ33 |  |  |  | 0.730 |  |  |  |  |
| SBQ54_imp |  |  |  | 0.620 |  |  |  |  |
| Subs1 |  |  |  |  | 0.586 |  |  |  |
| Subs2 |  |  |  |  | 0.728 |  |  |  |
| Subs3 |  |  |  |  | 0.531 |  |  |  |
| Subs4 |  |  |  |  | 0.682 |  |  |  |
| Subs7_imp |  |  |  |  | 0.529 |  |  |  |
| Subs5_imp |  |  |  |  | 0.815 |  |  |  |
| SBQ76 |  |  |  |  |  | 0.569 |  |  |
| SBQ79 | 0.302 |  |  |  |  | 0.567 |  |  |
| SBQ53_imp |  |  |  |  |  |  | 0.553 |  |
| SBQ67 |  |  |  |  |  |  |  | 0.616 |
| SBQ68 |  |  |  |  |  |  |  | 0.693 |
| SBQ63 | 0.421 |  |  |  |  |  |  |  |
| SBQ82 | 0.359 |  |  |  |  |  |  |  |
| SBQ01 | 0.465 |  |  |  |  |  | 0.319 |  |
| SBQ62 | 0.396 |  |  |  |  |  |  |  |
| SBQ71 |  |  |  |  |  | 0.306 |  |  |
| SBQ10 |  | 0.395 |  |  |  |  |  |  |
| SBQ73 |  | 0.432 |  |  |  |  |  |  |
| SBQ55 |  |  |  |  |  |  | 0.335 |  |
| SBQ61 |  |  | 0.418 |  |  |  |  |  |
| SBQ65 |  |  |  |  |  |  |  |  |
| SBQ80 |  |  |  |  |  | 0.419 |  |  |
| SBQ77 |  |  |  |  |  | 0.444 |  |  |
| Subs6 |  |  |  |  | 0.409 |  |  |  |
| SBQ66_imp |  |  |  |  |  |  | 0.413 |  |
| SBQ72_imp |  |  |  | 0.406 |  |  | 0.350 |  |
| ABQ51_imp |  |  | 0.335 |  |  |  |  |  |
| SBQ37_imp |  |  | 0.370 | 0.334 |  |  |  |  |
| SBQ78_imp |  |  |  |  |  |  |  |  |
| SBQ75_imp |  |  |  |  |  | 0.463 |  |  |

| **Table S2. Labelling the 7 Factors Based on Item Contents** | | |
| --- | --- | --- |
| Labels/Contents | Estimate (CFA) | P(>\|z\|) |
| **Internalising problems** |  |  |
| SBQ08-INTERN-unhappy | 0.797 | 0.000 |
| SBQ64-INTERN-felt alone | 0.792 | 0.000 |
| SBQ05-INTERN-sad without reason | 0.714 | 0.000 |
| SBQ85-INTERN-felt like did everything wrong | 0.844 | 0.000 |
| SBQ83-INTERN-felt useless | 0.856 | 0.000 |
| SBQ84-INTERN-hated myself | 0.860 | 0.000 |
| SBQ01-INTERN-cried | 0.769 | 0.000 |
| SBQ03-INTERN-fear | 0.752 | 0.000 |
| SBQ04-INTERN-worried | 0.793 | 0.000 |
| SBQ81-INTERN-could not enjoy | 0.729 | 0.000 |
| SBQ63-INTERN-bored | 0.518 | 0.000 |
| SBQ82-INTERN-tired, did nothing | 0.649 | 0.000 |
| SBQ62-INTERN-could not doze off | 0.598 | 0.000 |
| SBQ79-PSYCHOSIS-thoughts as if not my own | 0.165 | 0.000 |
| **ADHD** |  |  |
| SBQ15-ADHD-easily distracted | 0.721 | 0.000 |
| SBQ16-ADHD-difficulties to concentrate | 0.791 | 0.000 |
| SBQ74-ADHD-forgetful | 0.703 | 0.000 |
| SBQ12-ADHD-restless | 0.669 | 0.000 |
| SBQ17-ADHD-inattentive | 0.781 | 0.000 |
| SBQ13-ADHD-hectic and fidgety | 0.743 | 0.000 |
| SBQ14-ADHD-could not settle for longer | 0.686 | 0.000 |
| SBQ10-ADHD-did things without thinking | 0.594 | 0.000 |
| SBQ73-ADHD-restless inside | 0.742 | 0.000 |
| **Physical aggression** |  |  |
| SBQ34-PHYSAGGR-violent attack | 0.916 | 0.000 |
| SBQ35-PHYSAGGR-hit, bite, kick others | 0.931 | 0.000 |
| SBQ33-PHYSAGGR-engage in brawl | 0.853 | 0.000 |
| SBQ54-REAGGR-aggressive when taken st | 0.763 | 0.000 |
| SBQ72-REAGGR-aggressive when insulted | 0.374 | 0.000 |
| SBQ37-PROAGGR-threat others to get st | 0.439 | 0.000 |
| **Indirect and proactive aggression** |  |  |
| SBQ21-INDAGGR-bad things behind back | 0.559 | 0.000 |
| SBQ52-PROAGGR-scare to force others | 0.800 | 0.000 |
| SBQ19-INDAGGR-incite others to dislik | 0.645 | 0.000 |
| SBQ22-INDAGGR-active exclusion | 0.687 | 0.000 |
| SBQ23-INDAGGR-told secrets when mad at so | 0.659 | 0.000 |
| SBQ51-PROAGGR-boss others around | 0.597 | 0.000 |
| SBQ61-PROAGGR-humiliated others | 0.828 | 0.000 |
| SBQ37-PROAGGR-threat others to get st | 0.504 | 0.000 |
| **Substance use** |  |  |
| Subs 1-INCID12M-cannabidiol | 0.695 | 0.000 |
| Subs 2-INCID12M-(Met-)Amphetamine | 0.898 | 0.000 |
| Subs 3-INCID12M-LSD, Psilocybin | 0.833 | 0.000 |
| Subs 4-INCID12M-Cocaine | 0.891 | 0.000 |
| Subs 5-INCID12M-XTC | 0.924 | 0.000 |
| Subs 6-INCID12M-cough syrup,pastilles,drops w codeine | 0.627 | 0.000 |
| Subs 7-INCID12M-THC | 0.745 | 0.000 |
| **Psychosis** |  |  |
| SBQ76-PSYCHOSIS-heard voices when alone | 0.711 | 0.000 |
| SBQ77-PSYCHOSIS-felt persecuted | 0.687 | 0.000 |
| SBQ75-PSYCHOSIS-saw things other people could not see | 0.464 | 0.000 |
| SBQ79-PSYCHOSIS-thoughts as if not my own | 0.644 | 0.000 |
| SBQ71-INTERN-self-injury | 0.875 | 0.000 |
| SBQ80-PSYCHOSIS-under control of force/power | 0.732 | 0.000 |
| **Reactive Aggression** |  |  |
| SBQ53-REAGGR-aggressive when teased | 0.770 | 0.000 |
| SBQ55-REAGGR-mad when not getting st | 0.744 | 0.000 |
| SBQ66-OPAGGR-yell at parent | 0.692 | 0.000 |
| SBQ01-INTERN-cried | -0.200 | 0.000 |
| SBQ72-REAGGR-aggressive when insulted | 0.522 | 0.000 |

| **Table S3. Pearson correlations in the validation sample (N = 587)** | | | | | | | | |
| --- | --- | --- | --- | --- | --- | --- | --- | --- |
|  | Int | ADHD | PhyAgg | IndProAgg | Subs | Psychosis | ReAgg | Connectedness |
| ADHD | .637^**^ | _ |  |  |  |  |  |  |
| PhyAgg | .149^**^ | .228^**^ | _ |  |  |  |  |  |
| IndProAgg | .169^**^ | .261^**^ | .556^**^ | _ |  |  |  |  |
| Subs | .116^**^ | .235^**^ | .186^**^ | .185^**^ | _ |  |  |  |
| Psychosis | .584^**^ | .351^**^ | .224^**^ | .295^**^ | 0.066 | _ |  |  |
| ReAgg | .573^**^ | .524^**^ | .458^**^ | .366^**^ | 0.079 | .393^**^ | _ |  |
| Connectedness | -.224^**^ | -.145^**^ | -0.031 | -.110^**^ | -.130^**^ | -.195^**^ | -.161^**^ | _ |
| Valence | -.385^**^ | -.265^**^ | -0.050 | -0.049 | -.134^**^ | -.150^**^ | -.195^**^ | .158^**^ |

*Note. Factors are defined by the item groupings identified in the exploratory factor analysis conducted in the calibration subsample (N=593).*

| **Table S4. Model Fit for All the Models (N=587)** | | | | |
| --- | --- | --- | --- | --- |
| Model | CFI | TLI | RMSEA | SRMR |
| CFA | 0.954 | 0.951 | 0.037 | 0.073 |
| CFA SEM | 0.954 | 0.951 | 0.036 | 0.071 |
| Symmetry Bifactor | 0.912 | 0.904 | 0.052 | 0.096 |
| Symmetry Bifactor SEM | 0.913 | 0.904 | 0.050 | 0.093 |
| S-1 Bifactor | 0.956 | 0.953 | 0.036 | 0.070 |
| **S-1 Bifactor SEM** | **0.956** | **0.952** | **0.035** | **0.069** |
| Substance SEM | 0.982 | 0.975 | 0.062 | 0.061 |
| ADHD SEM | 0.931 | 0.911 | 0.126 | 0.060 |
| Internalising SEM | 0.966 | 0.961 | 0.079 | 0.049 |
| Physical aggression SEM | 0.989 | 0.984 | 0.055 | 0.046 |
| Indirect and proactive aggression SEM | 0.946 | 0.928 | 0.067 | 0.068 |
| Psychosis SEM | 0.960 | 0.940 | 0.056 | 0.064 |
| Reactive aggression SEM | 0.969 | 0.949 | 0.071 | 0.049 |

| **Table S5. CFA results (N=587)** | | | | | | |
| --- | --- | --- | --- | --- | --- | --- |
| Latent Variables: | Estimate | Std.Err | z-value | P(>\|z\|) | Std.lv | Std.all |
| Int =~ |  |  |  |  |  |  |
| SBQ08 | 0.797 | 0.019 | 41.848 | 0.000 | 0.797 | 0.797 |
| SBQ64 | 0.792 | 0.018 | 43.610 | 0.000 | 0.792 | 0.792 |
| SBQ05 | 0.714 | 0.024 | 29.711 | 0.000 | 0.714 | 0.714 |
| SBQ85 | 0.844 | 0.015 | 55.928 | 0.000 | 0.844 | 0.844 |
| SBQ83 | 0.856 | 0.016 | 53.325 | 0.000 | 0.856 | 0.856 |
| SBQ84 | 0.860 | 0.018 | 48.745 | 0.000 | 0.860 | 0.860 |
| SBQ03 | 0.752 | 0.024 | 31.556 | 0.000 | 0.752 | 0.752 |
| SBQ04 | 0.793 | 0.018 | 44.361 | 0.000 | 0.793 | 0.793 |
| SBQ81 | 0.729 | 0.022 | 33.129 | 0.000 | 0.729 | 0.729 |
| SBQ63 | 0.518 | 0.031 | 16.697 | 0.000 | 0.518 | 0.518 |
| SBQ82 | 0.649 | 0.028 | 23.326 | 0.000 | 0.649 | 0.649 |
| SBQ01 | 0.769 | 0.041 | 18.596 | 0.000 | 0.769 | 0.769 |
| SBQ62 | 0.598 | 0.029 | 20.766 | 0.000 | 0.598 | 0.598 |
| SBQ79 | 0.165 | 0.101 | 1.625 | 0.104 | 0.165 | 0.165 |
| ADHD =~ |  |  |  |  |  |  |
| SBQ15 | 0.721 | 0.023 | 31.773 | 0.000 | 0.721 | 0.721 |
| SBQ16 | 0.791 | 0.020 | 38.710 | 0.000 | 0.791 | 0.791 |
| SBQ74 | 0.703 | 0.026 | 27.263 | 0.000 | 0.703 | 0.703 |
| SBQ12 | 0.669 | 0.026 | 25.977 | 0.000 | 0.669 | 0.669 |
| SBQ17 | 0.781 | 0.021 | 37.158 | 0.000 | 0.781 | 0.781 |
| SBQ13 | 0.743 | 0.023 | 32.157 | 0.000 | 0.743 | 0.743 |
| SBQ14 | 0.686 | 0.025 | 27.913 | 0.000 | 0.686 | 0.686 |
| SBQ10 | 0.594 | 0.030 | 19.761 | 0.000 | 0.594 | 0.594 |
| SBQ73 | 0.742 | 0.025 | 29.447 | 0.000 | 0.742 | 0.742 |
| PhyAgg =~ |  |  |  |  |  |  |
| SBQ34 | 0.916 | 0.021 | 43.710 | 0.000 | 0.916 | 0.916 |
| SBQ35 | 0.931 | 0.025 | 37.179 | 0.000 | 0.931 | 0.931 |
| SBQ33 | 0.853 | 0.031 | 27.142 | 0.000 | 0.853 | 0.853 |
| SBQ54 | 0.763 | 0.033 | 22.993 | 0.000 | 0.763 | 0.763 |
| SBQ72 | 0.374 | 0.046 | 8.146 | 0.000 | 0.374 | 0.374 |
| SBQ37 | 0.439 | 0.097 | 4.538 | 0.000 | 0.439 | 0.439 |
| IndProAgg =~ |  |  |  |  |  |  |
| SBQ21 | 0.559 | 0.043 | 12.979 | 0.000 | 0.559 | 0.559 |
| SBQ52 | 0.800 | 0.037 | 21.681 | 0.000 | 0.800 | 0.800 |
| SBQ19 | 0.645 | 0.041 | 15.838 | 0.000 | 0.645 | 0.645 |
| SBQ22 | 0.687 | 0.044 | 15.546 | 0.000 | 0.687 | 0.687 |
| SBQ23 | 0.659 | 0.059 | 11.254 | 0.000 | 0.659 | 0.659 |
| SBQ61 | 0.828 | 0.035 | 23.887 | 0.000 | 0.828 | 0.828 |
| SBQ51 | 0.597 | 0.044 | 13.614 | 0.000 | 0.597 | 0.597 |
| SBQ37 | 0.504 | 0.107 | 4.717 | 0.000 | 0.504 | 0.504 |
| Subs =~ |  |  |  |  |  |  |
| SUBUSE1 | 0.695 | 0.045 | 15.506 | 0.000 | 0.695 | 0.695 |
| SUBUSE2 | 0.898 | 0.025 | 35.574 | 0.000 | 0.898 | 0.898 |
| SUBUSE3 | 0.833 | 0.034 | 24.514 | 0.000 | 0.833 | 0.833 |
| SUBUSE4 | 0.891 | 0.029 | 31.252 | 0.000 | 0.891 | 0.891 |
| SUBUSE7 | 0.745 | 0.037 | 20.382 | 0.000 | 0.745 | 0.745 |
| SUBUSE5 | 0.924 | 0.027 | 34.808 | 0.000 | 0.924 | 0.924 |
| SUBUSE6 | 0.627 | 0.057 | 10.920 | 0.000 | 0.627 | 0.627 |
| Psychosis =~ |  |  |  |  |  |  |
| SBQ76 | 0.711 | 0.050 | 14.320 | 0.000 | 0.711 | 0.711 |
| SBQ77 | 0.644 | 0.108 | 5.952 | 0.000 | 0.644 | 0.644 |
| SBQ75 | 0.875 | 0.055 | 15.874 | 0.000 | 0.875 | 0.875 |
| SBQ79 | 0.732 | 0.040 | 18.267 | 0.000 | 0.732 | 0.732 |
| SBQ71 | 0.687 | 0.048 | 14.221 | 0.000 | 0.687 | 0.687 |
| SBQ80 | 0.464 | 0.066 | 7.014 | 0.000 | 0.464 | 0.464 |
| ReAgg =~ |  |  |  |  |  |  |
| SBQ53 | 0.770 | 0.030 | 25.460 | 0.000 | 0.770 | 0.770 |
| SBQ55 | 0.744 | 0.039 | 18.915 | 0.000 | 0.744 | 0.744 |
| SBQ66 | 0.692 | 0.037 | 18.601 | 0.000 | 0.692 | 0.692 |
| SBQ01 | -0.200 | 0.056 | -3.564 | 0.000 | -0.200 | -0.200 |
| SBQ72 | 0.522 | 0.050 | 10.426 | 0.000 | 0.522 | 0.522 |
|  |  |  |  |  |  |  |
| Covariances: | Estimate | Std.Err | z-value | P(>\|z\|) | Std.lv | Std.all |
| Int ~~ |  |  |  |  |  |  |
| ADHD | 0.701 | 0.023 | 29.913 | 0.000 | 0.701 | 0.701 |
| PhyAgg | 0.081 | 0.051 | 1.595 | 0.111 | 0.081 | 0.081 |
| IndProAgg | 0.237 | 0.045 | 5.307 | 0.000 | 0.237 | 0.237 |
| Subs | 0.173 | 0.051 | 3.404 | 0.001 | 0.173 | 0.173 |
| Psychosis | 0.719 | 0.038 | 18.859 | 0.000 | 0.719 | 0.719 |
| ReAgg | 0.515 | 0.039 | 13.330 | 0.000 | 0.515 | 0.515 |
| ADHD ~~ |  |  |  |  |  |  |
| PhyAgg | 0.168 | 0.050 | 3.337 | 0.001 | 0.168 | 0.168 |
| IndProAgg | 0.340 | 0.042 | 8.109 | 0.000 | 0.340 | 0.340 |
| Subs | 0.322 | 0.050 | 6.460 | 0.000 | 0.322 | 0.322 |
| Psychosis | 0.468 | 0.047 | 9.945 | 0.000 | 0.468 | 0.468 |
| ReAgg | 0.571 | 0.035 | 16.260 | 0.000 | 0.571 | 0.571 |
| PhyAgg ~~ |  |  |  |  |  |  |
| IndProAgg | 0.683 | 0.038 | 17.799 | 0.000 | 0.683 | 0.683 |
| Subs | 0.322 | 0.059 | 5.441 | 0.000 | 0.322 | 0.322 |
| Psychosis | 0.267 | 0.066 | 4.014 | 0.000 | 0.267 | 0.267 |
| ReAgg | 0.450 | 0.052 | 8.675 | 0.000 | 0.450 | 0.450 |
| IndProAgg ~~ |  |  |  |  |  |  |
| Subs | 0.289 | 0.061 | 4.768 | 0.000 | 0.289 | 0.289 |
| Psychosis | 0.452 | 0.057 | 7.976 | 0.000 | 0.452 | 0.452 |
| ReAgg | 0.559 | 0.039 | 14.228 | 0.000 | 0.559 | 0.559 |
| Subs ~~ |  |  |  |  |  |  |
| Psychosis | 0.151 | 0.068 | 2.225 | 0.026 | 0.151 | 0.151 |
| ReAgg | 0.134 | 0.061 | 2.184 | 0.029 | 0.134 | 0.134 |
| Psychosis ~~ |  |  |  |  |  |  |
| ReAgg | 0.424 | 0.047 | 8.975 | 0.000 | 0.424 | 0.424 |

| **Table S6. Symmetry bifactor SEM with 95%CL (N=587)** | | | | | | |
| --- | --- | --- | --- | --- | --- | --- |
| Latent Variables: | est.std | Std.Err | z-value | P(>\|z\|) | ci.lower | ci.upper |
| p-factor =~ |  |  |  |  |  |  |
| SBQ08 | 0.577 | 0.034 | 16.862 | 0.000 | 0.510 | 0.644 |
| SBQ64 | 0.560 | 0.035 | 15.907 | 0.000 | 0.491 | 0.629 |
| SBQ05 | 0.544 | 0.036 | 15.099 | 0.000 | 0.474 | 0.615 |
| SBQ85 | 0.643 | 0.032 | 20.080 | 0.000 | 0.580 | 0.705 |
| SBQ83 | 0.673 | 0.032 | 20.872 | 0.000 | 0.610 | 0.736 |
| SBQ84 | 0.673 | 0.033 | 20.403 | 0.000 | 0.608 | 0.737 |
| SBQ03 | 0.591 | 0.035 | 16.979 | 0.000 | 0.523 | 0.660 |
| SBQ04 | 0.594 | 0.032 | 18.678 | 0.000 | 0.531 | 0.656 |
| SBQ81 | 0.586 | 0.033 | 17.823 | 0.000 | 0.521 | 0.650 |
| SBQ63 | 0.492 | 0.034 | 14.324 | 0.000 | 0.425 | 0.560 |
| SBQ82 | 0.594 | 0.033 | 17.879 | 0.000 | 0.529 | 0.659 |
| SBQ01 | 0.421 | 0.038 | 11.190 | 0.000 | 0.348 | 0.495 |
| SBQ62 | 0.479 | 0.033 | 14.478 | 0.000 | 0.414 | 0.544 |
| SBQ15 | 0.515 | 0.039 | 13.209 | 0.000 | 0.437 | 0.618 |
| SBQ16 | 0.594 | 0.037 | 16.040 | 0.000 | 0.438 | 0.591 |
| SBQ74 | 0.604 | 0.034 | 17.859 | 0.000 | 0.522 | 0.667 |
| SBQ12 | 0.609 | 0.032 | 19.187 | 0.000 | 0.538 | 0.671 |
| SBQ17 | 0.661 | 0.032 | 20.647 | 0.000 | 0.547 | 0.671 |
| SBQ13 | 0.675 | 0.029 | 23.114 | 0.000 | 0.598 | 0.724 |
| SBQ14 | 0.541 | 0.037 | 14.457 | 0.000 | 0.618 | 0.732 |
| SBQ10 | 0.538 | 0.034 | 15.984 | 0.000 | 0.468 | 0.615 |
| SBQ73 | 0.730 | 0.031 | 23.926 | 0.000 | 0.472 | 0.604 |
| SBQ34 | 0.295 | 0.055 | 5.392 | 0.000 | 0.670 | 0.790 |
| SBQ35 | 0.343 | 0.054 | 6.377 | 0.000 | 0.188 | 0.403 |
| SBQ33 | 0.187 | 0.062 | 3.004 | 0.003 | 0.238 | 0.449 |
| SBQ54 | 0.294 | 0.054 | 5.477 | 0.000 | 0.065 | 0.309 |
| SBQ72 | 0.443 | 0.040 | 10.993 | 0.000 | 0.189 | 0.399 |
| SBQ21 | 0.260 | 0.046 | 5.612 | 0.000 | 0.364 | 0.522 |
| SBQ52 | 0.401 | 0.050 | 7.987 | 0.000 | 0.265 | 0.522 |
| SBQ19 | 0.309 | 0.049 | 6.323 | 0.000 | 0.169 | 0.350 |
| SBQ22 | 0.297 | 0.055 | 5.431 | 0.000 | 0.302 | 0.499 |
| SBQ23 | 0.390 | 0.061 | 6.436 | 0.000 | 0.213 | 0.404 |
| SBQ61 | 0.408 | 0.048 | 8.410 | 0.000 | 0.190 | 0.404 |
| SBQ51 | 0.288 | 0.046 | 6.198 | 0.000 | 0.272 | 0.509 |
| SBQ37 | 0.394 | 0.065 | 6.011 | 0.000 | 0.313 | 0.503 |
| Subs1 | 0.180 | 0.054 | 3.357 | 0.001 | 0.197 | 0.379 |
| Subs2 | 0.343 | 0.079 | 4.328 | 0.000 | 0.075 | 0.286 |
| Subs3 | 0.178 | 0.074 | 2.396 | 0.017 | 0.188 | 0.499 |
| Subs4 | 0.327 | 0.064 | 5.128 | 0.000 | 0.032 | 0.323 |
| Subs7 | 0.207 | 0.047 | 4.375 | 0.000 | 0.202 | 0.452 |
| Subs5 | 0.305 | 0.067 | 4.523 | 0.000 | 0.114 | 0.300 |
| Subs6 | 0.261 | 0.066 | 3.975 | 0.000 | 0.173 | 0.437 |
| SBQ76 | 0.521 | 0.054 | 9.562 | 0.000 | 0.132 | 0.389 |
| SBQ79 | 0.528 | 0.046 | 11.409 | 0.000 | 0.414 | 0.628 |
| SBQ71 | 0.719 | 0.049 | 14.551 | 0.000 | 0.623 | 0.816 |
| SBQ80 | 0.564 | 0.043 | 12.997 | 0.000 | 0.479 | 0.649 |
| SBQ77 | 0.516 | 0.051 | 10.090 | 0.000 | 0.416 | 0.616 |
| SBQ75 | 0.282 | 0.064 | 4.392 | 0.000 | 0.156 | 0.407 |
| SBQ53 | 0.530 | 0.034 | 15.544 | 0.000 | 0.463 | 0.597 |
| SBQ55 | 0.494 | 0.046 | 10.857 | 0.000 | 0.405 | 0.583 |
| SBQ66 | 0.491 | 0.038 | 13.022 | 0.000 | 0.417 | 0.565 |
| Int =~ |  |  |  |  |  |  |
| SBQ08 | 0.577 | 0.033 | 17.686 | 0.000 | 0.513 | 0.641 |
| SBQ64 | 0.598 | 0.032 | 18.414 | 0.000 | 0.535 | 0.662 |
| SBQ05 | 0.467 | 0.039 | 12.056 | 0.000 | 0.391 | 0.543 |
| SBQ85 | 0.556 | 0.034 | 16.438 | 0.000 | 0.489 | 0.622 |
| SBQ83 | 0.531 | 0.037 | 14.345 | 0.000 | 0.459 | 0.604 |
| SBQ84 | 0.535 | 0.038 | 14.057 | 0.000 | 0.460 | 0.609 |
| SBQ03 | 0.455 | 0.040 | 11.287 | 0.000 | 0.376 | 0.534 |
| SBQ04 | 0.540 | 0.033 | 16.558 | 0.000 | 0.476 | 0.604 |
| SBQ81 | 0.436 | 0.038 | 11.355 | 0.000 | 0.361 | 0.511 |
| SBQ63 | 0.143 | 0.044 | 3.250 | 0.001 | 0.057 | 0.229 |
| SBQ82 | 0.227 | 0.044 | 5.215 | 0.000 | 0.142 | 0.312 |
| SBQ01 | 0.530 | 0.036 | 14.673 | 0.000 | 0.459 | 0.601 |
| SBQ62 | 0.338 | 0.040 | 8.387 | 0.000 | 0.259 | 0.417 |
| SBQ79 | 0.356 | 0.059 | 6.052 | 0.000 | 0.241 | 0.472 |
| ADHD =~ |  |  |  |  |  |  |
| SBQ15 | 0.636 | 0.036 | 17.857 | 0.000 | 0.566 | 0.706 |
| SBQ16 | 0.635 | 0.037 | 17.029 | 0.000 | 0.562 | 0.708 |
| SBQ74 | 0.345 | 0.045 | 7.677 | 0.000 | 0.257 | 0.434 |
| SBQ12 | 0.218 | 0.048 | 4.577 | 0.000 | 0.124 | 0.311 |
| SBQ17 | 0.413 | 0.041 | 9.976 | 0.000 | 0.332 | 0.494 |
| SBQ13 | 0.251 | 0.044 | 5.695 | 0.000 | 0.165 | 0.338 |
| SBQ14 | 0.478 | 0.044 | 10.800 | 0.000 | 0.391 | 0.565 |
| SBQ10 | 0.213 | 0.046 | 4.674 | 0.000 | 0.124 | 0.303 |
| SBQ73 | 0.022 | 0.053 | 0.415 | 0.678 | -0.082 | 0.126 |
| PhyAgg =~ |  |  |  |  |  |  |
| SBQ34 | 0.879 | 0.029 | 30.662 | 0.000 | 0.823 | 0.935 |
| SBQ35 | 0.841 | 0.030 | 27.893 | 0.000 | 0.782 | 0.900 |
| SBQ33 | 0.888 | 0.028 | 32.261 | 0.000 | 0.834 | 0.942 |
| SBQ54 | 0.668 | 0.038 | 17.807 | 0.000 | 0.595 | 0.742 |
| SBQ72 | 0.471 | 0.042 | 11.113 | 0.000 | 0.388 | 0.554 |
| SBQ37 | 0.712 | 0.046 | 15.375 | 0.000 | 0.621 | 0.803 |
| IndProAgg =~ |  |  |  |  |  |  |
| SBQ21 | 0.553 | 0.041 | 13.452 | 0.000 | 0.472 | 0.634 |
| SBQ52 | 0.639 | 0.041 | 15.458 | 0.000 | 0.558 | 0.720 |
| SBQ19 | 0.645 | 0.042 | 15.416 | 0.000 | 0.563 | 0.727 |
| SBQ22 | 0.699 | 0.045 | 15.426 | 0.000 | 0.611 | 0.788 |
| SBQ23 | 0.509 | 0.060 | 8.497 | 0.000 | 0.392 | 0.626 |
| SBQ61 | 0.667 | 0.043 | 15.357 | 0.000 | 0.582 | 0.752 |
| SBQ51 | 0.479 | 0.049 | 9.693 | 0.000 | 0.382 | 0.576 |
| SBQ37 | 0.634 | 0.057 | 11.094 | 0.000 | 0.522 | 0.746 |
| Subs =~ |  |  |  |  |  |  |
| Subs1 | 0.695 | 0.042 | 16.685 | 0.000 | 0.614 | 0.777 |
| Subs2 | 0.825 | 0.035 | 23.338 | 0.000 | 0.756 | 0.895 |
| Subs3 | 0.853 | 0.027 | 31.758 | 0.000 | 0.800 | 0.905 |
| Subs4 | 0.807 | 0.032 | 25.543 | 0.000 | 0.745 | 0.869 |
| Subs7 | 0.744 | 0.038 | 19.480 | 0.000 | 0.669 | 0.819 |
| Subs5 | 0.870 | 0.030 | 28.571 | 0.000 | 0.810 | 0.930 |
| Subs6 | 0.510 | 0.052 | 9.830 | 0.000 | 0.409 | 0.612 |
| Psychosis =~ |  |  |  |  |  |  |
| SBQ76 | 0.633 | 0.061 | 10.342 | 0.000 | 0.513 | 0.753 |
| SBQ79 | 0.461 | 0.063 | 7.355 | 0.000 | 0.338 | 0.584 |
| SBQ71 | 0.237 | 0.080 | 2.946 | 0.003 | 0.079 | 0.394 |
| SBQ80 | 0.464 | 0.056 | 8.232 | 0.000 | 0.354 | 0.575 |
| SBQ77 | 0.511 | 0.065 | 7.887 | 0.000 | 0.384 | 0.637 |
| SBQ75 | 0.656 | 0.075 | 8.719 | 0.000 | 0.509 | 0.804 |
| ReAgg =~ |  |  |  |  |  |  |
| SBQ53 | 0.561 | 0.045 | 12.334 | 0.000 | 0.472 | 0.650 |
| SBQ55 | 0.599 | 0.050 | 12.082 | 0.000 | 0.501 | 0.696 |
| SBQ66 | 0.459 | 0.040 | 11.496 | 0.000 | 0.381 | 0.537 |
| SBQ01 | 0.053 | 0.050 | 1.051 | 0.293 | -0.045 | 0.150 |
| SBQ72 | 0.578 | 0.048 | 12.141 | 0.000 | 0.485 | 0.672 |
|  |  |  |  |  |  |  |
| Covariances: | est.std | Std.Err | z-value | P(>\|z\|) | ci.lower | ci.upper |
| Self-valence ~~ |  |  |  |  |  |  |
| p-factor | -0.358 | 0.040 | -8.918 | 0.000 | -0.436 | -0.279 |
| Int | -0.244 | 0.046 | -5.293 | 0.000 | -0.335 | -0.154 |
| ADHD | -0.007 | 0.053 | -0.133 | 0.894 | -0.112 | 0.098 |
| Psychosis | 0.140 | 0.067 | 2.087 | 0.037 | 0.009 | 0.272 |
| Subs | -0.037 | 0.053 | -0.708 | 0.479 | -0.140 | 0.066 |
| PhyAgg | 0.061 | 0.049 | 1.252 | 0.211 | -0.035 | 0.157 |
| IndProAgg | 0.104 | 0.053 | 1.970 | 0.049 | 0.001 | 0.207 |
| ReAgg | 0.044 | 0.049 | 0.906 | 0.365 | -0.052 | 0.140 |
| Self-connectedness ~~ |  |  |  |  |  |  |
| p-factor | -0.173 | 0.046 | -3.771 | 0.000 | -0.263 | -0.083 |
| Int | -0.142 | 0.045 | -3.189 | 0.001 | -0.229 | -0.055 |
| ADHD | -0.010 | 0.048 | -0.209 | 0.835 | -0.105 | 0.084 |
| Psychosis | -0.168 | 0.059 | -2.859 | 0.004 | -0.283 | -0.053 |
| Subs | -0.116 | 0.053 | -2.193 | 0.028 | -0.219 | -0.012 |
| PhyAgg | 0.079 | 0.046 | 1.704 | 0.088 | -0.012 | 0.169 |
| IndProAgg | -0.051 | 0.051 | -0.998 | 0.318 | -0.150 | 0.049 |
| ReAgg | -0.045 | 0.050 | -0.905 | 0.365 | -0.142 | 0.052 |
| Self-valence ~~ |  |  |  |  |  |  |
| self-connectedness | 0.174 | 0.040 | 4.375 | 0.000 | 0.096 | 0.252 |
| Note: lavaan WARNING: some estimated ov variances are negative | | | | | | |


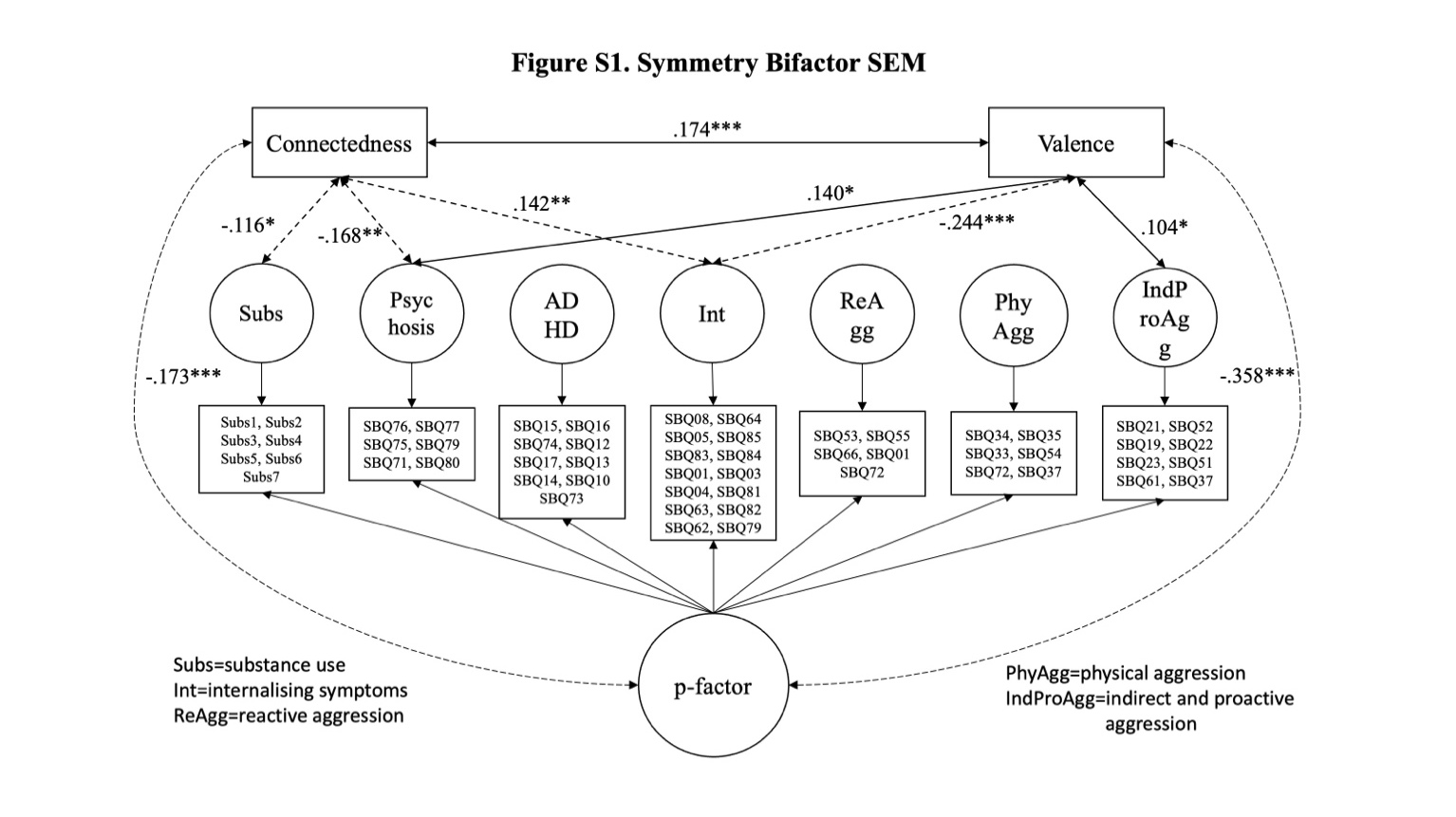


| **Table S7. S-1 bifactor correlation SEM with 95%CL (N=587)** | | | | | | |
| --- | --- | --- | --- | --- | --- | --- |
| Latent Variables: | est.std | Std.Err | z-value | P(>\|z\|) | ci.lower | ci.upper |
| Partially G-factor =~ |  |  |  |  |  |  |
| SBQ34 | 0.899 | 0.021 | 41.896 | 0.000 | 0.856 | 0.941 |
| SBQ35 | 0.905 | 0.025 | 36.703 | 0.000 | 0.856 | 0.953 |
| SBQ33 | 0.832 | 0.032 | 25.658 | 0.000 | 0.769 | 0.896 |
| SBQ54 | 0.741 | 0.033 | 22.713 | 0.000 | 0.677 | 0.805 |
| SBQ72 | 0.668 | 0.031 | 21.265 | 0.000 | 0.606 | 0.729 |
| SBQ37 | 0.815 | 0.040 | 20.372 | 0.000 | 0.736 | 0.893 |
| SBQ21 | 0.304 | 0.051 | 5.939 | 0.000 | 0.204 | 0.405 |
| SBQ52 | 0.679 | 0.037 | 18.491 | 0.000 | 0.607 | 0.750 |
| SBQ19 | 0.367 | 0.051 | 7.230 | 0.000 | 0.268 | 0.467 |
| SBQ22 | 0.472 | 0.053 | 8.953 | 0.000 | 0.368 | 0.575 |
| SBQ23 | 0.344 | 0.068 | 5.080 | 0.000 | 0.211 | 0.477 |
| SBQ61 | 0.713 | 0.034 | 21.176 | 0.000 | 0.647 | 0.779 |
| SBQ51 | 0.510 | 0.040 | 12.784 | 0.000 | 0.432 | 0.588 |
| SBQ53 | 0.424 | 0.042 | 10.069 | 0.000 | 0.341 | 0.506 |
| SBQ55 | 0.515 | 0.042 | 12.149 | 0.000 | 0.432 | 0.598 |
| SBQ66 | 0.321 | 0.047 | 6.864 | 0.000 | 0.229 | 0.413 |
| SBQ01 | -0.220 | 0.042 | -5.312 | 0.000 | -0.302 | -0.139 |
| Int =~ |  |  |  |  |  |  |
| SBQ08 | 0.798 | 0.019 | 42.082 | 0.000 | 0.761 | 0.835 |
| SBQ64 | 0.792 | 0.018 | 43.689 | 0.000 | 0.757 | 0.828 |
| SBQ05 | 0.715 | 0.024 | 29.911 | 0.000 | 0.668 | 0.761 |
| SBQ85 | 0.844 | 0.015 | 55.679 | 0.000 | 0.814 | 0.874 |
| SBQ83 | 0.857 | 0.016 | 53.511 | 0.000 | 0.826 | 0.889 |
| SBQ84 | 0.860 | 0.018 | 48.961 | 0.000 | 0.826 | 0.895 |
| SBQ03 | 0.750 | 0.024 | 31.323 | 0.000 | 0.703 | 0.797 |
| SBQ04 | 0.793 | 0.018 | 44.437 | 0.000 | 0.758 | 0.828 |
| SBQ81 | 0.731 | 0.022 | 33.513 | 0.000 | 0.689 | 0.774 |
| SBQ63 | 0.518 | 0.031 | 16.745 | 0.000 | 0.457 | 0.579 |
| SBQ82 | 0.649 | 0.028 | 23.485 | 0.000 | 0.595 | 0.704 |
| SBQ01 | 0.594 | 0.041 | 14.562 | 0.000 | 0.514 | 0.674 |
| SBQ62 | 0.598 | 0.029 | 20.820 | 0.000 | 0.542 | 0.654 |
| SBQ79 | 0.161 | 0.101 | 1.584 | 0.113 | -0.038 | 0.360 |
| ADHD =~ |  |  |  |  |  |  |
| SBQ15 | 0.721 | 0.023 | 31.660 | 0.000 | 0.676 | 0.765 |
| SBQ16 | 0.792 | 0.020 | 38.945 | 0.000 | 0.752 | 0.832 |
| SBQ74 | 0.703 | 0.026 | 27.278 | 0.000 | 0.653 | 0.754 |
| SBQ12 | 0.667 | 0.026 | 25.831 | 0.000 | 0.617 | 0.718 |
| SBQ17 | 0.781 | 0.021 | 37.090 | 0.000 | 0.740 | 0.822 |
| SBQ13 | 0.742 | 0.023 | 32.031 | 0.000 | 0.697 | 0.788 |
| SBQ14 | 0.686 | 0.025 | 27.787 | 0.000 | 0.637 | 0.734 |
| SBQ10 | 0.594 | 0.030 | 19.657 | 0.000 | 0.535 | 0.653 |
| SBQ73 | 0.744 | 0.025 | 29.327 | 0.000 | 0.694 | 0.794 |
| Subs =~ |  |  |  |  |  |  |
| Subs1 | 0.695 | 0.045 | 15.473 | 0.000 | 0.607 | 0.782 |
| Subs2 | 0.896 | 0.025 | 35.445 | 0.000 | 0.846 | 0.945 |
| Subs3 | 0.834 | 0.034 | 24.589 | 0.000 | 0.767 | 0.900 |
| Subs4 | 0.894 | 0.028 | 31.508 | 0.000 | 0.838 | 0.949 |
| Subs7 | 0.745 | 0.037 | 20.362 | 0.000 | 0.674 | 0.817 |
| Subs5 | 0.923 | 0.027 | 34.590 | 0.000 | 0.871 | 0.976 |
| Subs6 | 0.626 | 0.057 | 10.948 | 0.000 | 0.514 | 0.738 |
| Psychosis =~ |  |  |  |  |  |  |
| SBQ76 | 0.709 | 0.050 | 14.279 | 0.000 | 0.612 | 0.806 |
| SBQ79 | 0.650 | 0.108 | 6.011 | 0.000 | 0.438 | 0.862 |
| SBQ71 | 0.879 | 0.055 | 16.007 | 0.000 | 0.771 | 0.986 |
| SBQ80 | 0.728 | 0.040 | 18.152 | 0.000 | 0.650 | 0.807 |
| SBQ77 | 0.685 | 0.048 | 14.243 | 0.000 | 0.591 | 0.780 |
| SBQ75 | 0.462 | 0.066 | 6.991 | 0.000 | 0.333 | 0.592 |
| IndProAgg =~ |  |  |  |  |  |  |
| SBQ21 | 0.563 | 0.054 | 10.444 | 0.000 | 0.458 | 0.669 |
| SBQ52 | 0.379 | 0.063 | 6.017 | 0.000 | 0.256 | 0.503 |
| SBQ19 | 0.630 | 0.056 | 11.235 | 0.000 | 0.520 | 0.740 |
| SBQ22 | 0.535 | 0.063 | 8.515 | 0.000 | 0.412 | 0.659 |
| SBQ23 | 0.691 | 0.078 | 8.836 | 0.000 | 0.538 | 0.845 |
| SBQ61 | 0.347 | 0.062 | 5.626 | 0.000 | 0.226 | 0.468 |
| SBQ51 | 0.290 | 0.066 | 4.414 | 0.000 | 0.161 | 0.419 |
| SBQ37 | 0.284 | 0.077 | 3.699 | 0.000 | 0.134 | 0.435 |
| ReAgg =~ |  |  |  |  |  |  |
| SBQ53 | 0.663 | 0.042 | 15.607 | 0.000 | 0.580 | 0.746 |
| SBQ55 | 0.538 | 0.051 | 10.557 | 0.000 | 0.438 | 0.638 |
| SBQ66 | 0.644 | 0.047 | 13.696 | 0.000 | 0.552 | 0.736 |
| SBQ01 | 0.167 | 0.052 | 3.216 | 0.001 | 0.065 | 0.269 |
| SBQ72 | 0.405 | 0.048 | 8.377 | 0.000 | 0.310 | 0.500 |
|  |  |  |  |  |  |  |
| Covariances: | est.std | Std.Err | z-value | P(>\|z\|) | ci.lower | ci.upper |
| G-factor ~~ |  |  |  |  |  |  |
| IndProAgg | 0.000 |  |  |  | 0.000 | 0.000 |
| ReAgg | 0.000 |  |  |  | 0.000 | 0.000 |
| IndProAgg ~~ |  |  |  |  |  |  |
| ReAgg | 0.000 |  |  |  | 0.000 | 0.000 |
| Self-valence ~~ |  |  |  |  |  |  |
| G-factor | -0.125 | 0.050 | -2.529 | 0.011 | -0.223 | -0.028 |
| Int | -0.436 | 0.038 | -11.606 | 0.000 | -0.510 | -0.362 |
| ADHD | -0.301 | 0.040 | -7.488 | 0.000 | -0.380 | -0.222 |
| Psychosis | -0.220 | 0.054 | -4.035 | 0.000 | -0.327 | -0.113 |
| Subs | -0.143 | 0.052 | -2.743 | 0.006 | -0.246 | -0.041 |
| Self-connectedness ~~ |  |  |  |  |  |  |
| G-factor | -0.085 | 0.049 | -1.729 | 0.084 | -0.181 | 0.011 |
| Int | -0.226 | 0.041 | -5.502 | 0.000 | -0.307 | -0.145 |
| ADHD | -0.151 | 0.041 | -3.658 | 0.000 | -0.231 | -0.070 |
| Psychosis | -0.260 | 0.051 | -5.150 | 0.000 | -0.359 | -0.161 |
| Subs | -0.166 | 0.051 | -3.277 | 0.001 | -0.265 | -0.067 |
| Self-valence ~~ |  |  |  |  |  |  |
| self-connectedness | 0.174 | 0.040 | 4.375 | 0.000 | 0.096 | 0.252 |
| G-factor ~~ |  |  |  |  |  |  |
| Int | 0.111 | 0.050 | 2.219 | 0.026 | 0.013 | 0.209 |
| ADHD | 0.190 | 0.049 | 3.881 | 0.000 | 0.094 | 0.285 |
| Subs | 0.326 | 0.058 | 5.669 | 0.000 | 0.214 | 0.439 |
| Psychosis | 0.260 | 0.067 | 3.903 | 0.000 | 0.129 | 0.391 |
| Int ~~ |  |  |  |  |  |  |
| ADHD | 0.698 | 0.024 | 29.456 | 0.000 | 0.652 | 0.745 |
| Subs | 0.183 | 0.051 | 3.584 | 0.000 | 0.083 | 0.282 |
| Psychosis | 0.722 | 0.038 | 18.810 | 0.000 | 0.647 | 0.797 |
| IndProAgg | 0.231 | 0.062 | 3.748 | 0.000 | 0.110 | 0.352 |
| ReAgg | 0.523 | 0.049 | 10.653 | 0.000 | 0.426 | 0.619 |
| ADHD ~~ |  |  |  |  |  |  |
| Subs | 0.322 | 0.050 | 6.458 | 0.000 | 0.224 | 0.420 |
| Psychosis | 0.469 | 0.047 | 9.944 | 0.000 | 0.377 | 0.562 |
| IndProAgg | 0.291 | 0.057 | 5.152 | 0.000 | 0.181 | 0.402 |
| ReAgg | 0.546 | 0.046 | 11.822 | 0.000 | 0.456 | 0.637 |
| Subs ~~ |  |  |  |  |  |  |
| Psychosis | 0.151 | 0.068 | 2.222 | 0.026 | 0.018 | 0.285 |
| IndProAgg | 0.077 | 0.071 | 1.084 | 0.279 | -0.062 | 0.216 |
| ReAgg | -0.074 | 0.065 | -1.146 | 0.252 | -0.201 | 0.053 |
| Psychosis ~~ |  |  |  |  |  |  |
| IndProAgg | 0.383 | 0.064 | 6.003 | 0.000 | 0.258 | 0.508 |
| ReAgg | 0.349 | 0.062 | 5.585 | 0.000 | 0.226 | 0.471 |

**Appendix S1. SEM based on the multifactor CFA oblique model:** The results of the SEM based on the multifactor CFA oblique model achieved adequate loadings for all the factors except 2 items with cross-loadings. The model indices were above conventional standards (CFI= .954, TLI= .951, RMSEA= .036, and SRMR= .071). As shown in Table S8 and Figure S2: 1) future self-valence is significantly and negatively associated with internalising symptoms (*r* = -.430, *p*< .01, [95% CI = -0.504, -0.357]), ADHD (*r* = -.301, *p*< .001, [95% CI = -0.380, -0.222]), psychosis-like symptoms (*r* = -.220, *p*< .001, [95% CI = -0.326, -0.113]), reactive aggression (*r* = -.216, *p*< .001, [95% CI = -0.303, -0.129]) and substance use (*r* = -.143, *p*< .01, [95% CI = -0.246, -0.041]); 2) future self-connectedness is negatively associated with internalising symptoms (*r* = -.224, *p*< .001, [95% CI = -0.303, -0.144]), ADHD (*r* = -.151, *p*< .001, [95% CI = -0.231, -0.070]), psychosis-like symptoms (*r* = -.260, *p*< .001, [95% CI = -0.359, -0.161]), indirect and proactive aggression (*r* = -.133, *p*< .01, [95% CI = -0.230, -0.035]), reactive aggression (*r* = -.138, *p*< .01, [95% CI = -0.230, -0.047]) and substance use (*r* = -.166, *p*< .01, [95% CI = -0.265, -0.067]).

| **Table S8. CFA SEM with 95%CL (N=587)** | | | | | | |
| --- | --- | --- | --- | --- | --- | --- |
| Latent Variables: | est.std | Std.Err | z-value | P(>\|z\|) | ci.lower | ci.upper |
| Int =~ |  |  |  |  |  |  |
| SBQ08 | 0.798 | 0.019 | 42.009 | 0.000 | 0.761 | 0.835 |
| SBQ64 | 0.792 | 0.018 | 43.621 | 0.000 | 0.756 | 0.827 |
| SBQ05 | 0.714 | 0.024 | 29.938 | 0.000 | 0.668 | 0.761 |
| SBQ85 | 0.844 | 0.015 | 55.660 | 0.000 | 0.814 | 0.874 |
| SBQ83 | 0.857 | 0.016 | 53.444 | 0.000 | 0.825 | 0.888 |
| SBQ84 | 0.860 | 0.018 | 48.912 | 0.000 | 0.825 | 0.894 |
| SBQ03 | 0.749 | 0.024 | 31.360 | 0.000 | 0.703 | 0.796 |
| SBQ04 | 0.793 | 0.018 | 44.446 | 0.000 | 0.758 | 0.828 |
| SBQ81 | 0.731 | 0.022 | 33.433 | 0.000 | 0.688 | 0.773 |
| SBQ63 | 0.517 | 0.031 | 16.731 | 0.000 | 0.457 | 0.578 |
| SBQ82 | 0.649 | 0.028 | 23.468 | 0.000 | 0.595 | 0.703 |
| SBQ01 | 0.765 | 0.041 | 18.450 | 0.000 | 0.684 | 0.846 |
| SBQ62 | 0.598 | 0.029 | 20.821 | 0.000 | 0.542 | 0.654 |
| SBQ79 | 0.165 | 0.100 | 1.653 | 0.098 | -0.031 | 0.361 |
| ADHD =~ |  |  |  |  |  |  |
| SBQ15 | 0.721 | 0.023 | 31.655 | 0.000 | 0.676 | 0.765 |
| SBQ16 | 0.792 | 0.020 | 38.935 | 0.000 | 0.752 | 0.832 |
| SBQ74 | 0.703 | 0.026 | 27.276 | 0.000 | 0.653 | 0.754 |
| SBQ12 | 0.667 | 0.026 | 25.828 | 0.000 | 0.617 | 0.718 |
| SBQ17 | 0.781 | 0.021 | 37.066 | 0.000 | 0.740 | 0.822 |
| SBQ13 | 0.742 | 0.023 | 32.011 | 0.000 | 0.697 | 0.788 |
| SBQ14 | 0.685 | 0.025 | 27.795 | 0.000 | 0.637 | 0.734 |
| SBQ10 | 0.594 | 0.030 | 19.675 | 0.000 | 0.535 | 0.653 |
| SBQ73 | 0.744 | 0.025 | 29.331 | 0.000 | 0.694 | 0.794 |
| PhyAgg =~ |  |  |  |  |  |  |
| SBQ34 | 0.915 | 0.021 | 43.714 | 0.000 | 0.874 | 0.956 |
| SBQ35 | 0.930 | 0.025 | 37.147 | 0.000 | 0.881 | 0.979 |
| SBQ33 | 0.853 | 0.031 | 27.106 | 0.000 | 0.791 | 0.914 |
| SBQ54 | 0.763 | 0.033 | 22.999 | 0.000 | 0.698 | 0.828 |
| SBQ72 | 0.371 | 0.046 | 8.097 | 0.000 | 0.281 | 0.461 |
| SBQ37 | 0.457 | 0.095 | 4.830 | 0.000 | 0.272 | 0.643 |
| IndProAgg =~ |  |  |  |  |  |  |
| SBQ21 | 0.561 | 0.043 | 13.015 | 0.000 | 0.476 | 0.645 |
| SBQ52 | 0.799 | 0.037 | 21.575 | 0.000 | 0.727 | 0.872 |
| SBQ19 | 0.647 | 0.041 | 15.908 | 0.000 | 0.567 | 0.727 |
| SBQ22 | 0.684 | 0.044 | 15.505 | 0.000 | 0.598 | 0.771 |
| SBQ23 | 0.660 | 0.058 | 11.287 | 0.000 | 0.545 | 0.774 |
| SBQ61 | 0.829 | 0.035 | 23.991 | 0.000 | 0.761 | 0.897 |
| SBQ51 | 0.598 | 0.044 | 13.623 | 0.000 | 0.512 | 0.684 |
| SBQ37 | 0.485 | 0.105 | 4.602 | 0.000 | 0.279 | 0.692 |
| Subs =~ |  |  |  |  |  |  |
| Subs1 | 0.694 | 0.045 | 15.466 | 0.000 | 0.606 | 0.782 |
| Subs2 | 0.896 | 0.025 | 35.542 | 0.000 | 0.847 | 0.946 |
| Subs3 | 0.834 | 0.034 | 24.553 | 0.000 | 0.767 | 0.900 |
| Subs4 | 0.893 | 0.028 | 31.391 | 0.000 | 0.837 | 0.949 |
| Subs7 | 0.746 | 0.037 | 20.364 | 0.000 | 0.674 | 0.818 |
| Subs5 | 0.924 | 0.027 | 34.637 | 0.000 | 0.872 | 0.976 |
| Subs6 | 0.625 | 0.057 | 10.927 | 0.000 | 0.513 | 0.737 |
| Psychosis =~ |  |  |  |  |  |  |
| SBQ76 | 0.710 | 0.050 | 14.286 | 0.000 | 0.612 | 0.807 |
| SBQ77 | 0.644 | 0.107 | 6.038 | 0.000 | 0.435 | 0.854 |
| SBQ75 | 0.881 | 0.055 | 16.122 | 0.000 | 0.774 | 0.988 |
| SBQ79 | 0.729 | 0.040 | 18.164 | 0.000 | 0.650 | 0.808 |
| SBQ71 | 0.685 | 0.048 | 14.204 | 0.000 | 0.590 | 0.779 |
| SBQ80 | 0.464 | 0.066 | 7.018 | 0.000 | 0.334 | 0.593 |
| ReAgg =~ |  |  |  |  |  |  |
| SBQ53 | 0.770 | 0.030 | 25.300 | 0.000 | 0.710 | 0.829 |
| SBQ55 | 0.743 | 0.039 | 18.844 | 0.000 | 0.666 | 0.820 |
| SBQ66 | 0.692 | 0.037 | 18.636 | 0.000 | 0.620 | 0.765 |
| SBQ01 | -0.197 | 0.056 | -3.506 | 0.000 | -0.307 | -0.087 |
| SBQ72 | 0.524 | 0.050 | 10.471 | 0.000 | 0.426 | 0.623 |
|  |  |  |  |  |  |  |
| Covariances: | est.std | Std.Err | z-value | P(>\|z\|) | ci.lower | ci.upper |
| Self-valence ~~ |  |  |  |  |  |  |
| Int | -0.430 | 0.037 | -11.520 | 0.000 | -0.504 | -0.357 |
| ADHD | -0.301 | 0.040 | -7.488 | 0.000 | -0.380 | -0.222 |
| Psychosis | -0.220 | 0.054 | -4.036 | 0.000 | -0.326 | -0.113 |
| PhyAgg | -0.048 | 0.055 | -0.874 | 0.382 | -0.155 | 0.059 |
| IndProAgg | -0.078 | 0.051 | -1.515 | 0.130 | -0.179 | 0.023 |
| ReAgg | -0.216 | 0.044 | -4.878 | 0.000 | -0.303 | -0.129 |
| Subs | -0.143 | 0.052 | -2.743 | 0.006 | -0.246 | -0.041 |
| Self-connectedness ~~ |  |  |  |  |  |  |
| Int | -0.224 | 0.041 | -5.513 | 0.000 | -0.303 | -0.144 |
| ADHD | -0.151 | 0.041 | -3.658 | 0.000 | -0.231 | -0.070 |
| Psychosis | -0.260 | 0.051 | -5.144 | 0.000 | -0.359 | -0.161 |
| PhyAgg | 0.015 | 0.049 | 0.306 | 0.760 | -0.081 | 0.111 |
| IndProAgg | -0.133 | 0.050 | -2.663 | 0.008 | -0.230 | -0.035 |
| ReAgg | -0.138 | 0.047 | -2.960 | 0.003 | -0.230 | -0.047 |
| Subs | -0.166 | 0.051 | -3.277 | 0.001 | -0.265 | -0.067 |
| Self-valence ~~ |  |  |  |  |  |  |
| self-connectedness | 0.174 | 0.040 | 4.375 | 0.000 | 0.096 | 0.252 |
| Int ~~ |  |  |  |  |  |  |
| ADHD | 0.701 | 0.023 | 29.913 | 0.000 | 0.655 | 0.747 |
| PhyAgg | 0.081 | 0.051 | 1.602 | 0.109 | -0.018 | 0.181 |
| IndProAgg | 0.237 | 0.045 | 5.301 | 0.000 | 0.149 | 0.324 |
| Subs | 0.173 | 0.051 | 3.405 | 0.001 | 0.073 | 0.272 |
| Psychosis | 0.719 | 0.038 | 18.884 | 0.000 | 0.644 | 0.793 |
| ReAgg | 0.514 | 0.039 | 13.308 | 0.000 | 0.438 | 0.590 |
| ADHD ~~ |  |  |  |  |  |  |
| PhyAgg | 0.168 | 0.050 | 3.342 | 0.001 | 0.070 | 0.267 |
| IndProAgg | 0.339 | 0.042 | 8.103 | 0.000 | 0.257 | 0.421 |
| Subs | 0.322 | 0.050 | 6.458 | 0.000 | 0.224 | 0.420 |
| Psychosis | 0.468 | 0.047 | 9.941 | 0.000 | 0.376 | 0.561 |
| ReAgg | 0.571 | 0.035 | 16.239 | 0.000 | 0.502 | 0.640 |
| PhyAgg ~~ |  |  |  |  |  |  |
| IndProAgg | 0.682 | 0.038 | 17.773 | 0.000 | 0.607 | 0.758 |
| Subs | 0.322 | 0.059 | 5.447 | 0.000 | 0.206 | 0.438 |
| Psychosis | 0.267 | 0.066 | 4.014 | 0.000 | 0.137 | 0.397 |
| ReAgg | 0.451 | 0.052 | 8.687 | 0.000 | 0.349 | 0.553 |
| IndProAgg ~~ |  |  |  |  |  |  |
| Subs | 0.288 | 0.061 | 4.765 | 0.000 | 0.170 | 0.407 |
| Psychosis | 0.452 | 0.057 | 7.974 | 0.000 | 0.341 | 0.564 |
| ReAgg | 0.559 | 0.039 | 14.213 | 0.000 | 0.482 | 0.636 |
| Subs ~~ |  |  |  |  |  |  |
| Psychosis | 0.152 | 0.068 | 2.226 | 0.026 | 0.018 | 0.285 |
| ReAgg | 0.134 | 0.061 | 2.181 | 0.029 | 0.014 | 0.254 |
| Psychosis ~~ |  |  |  |  |  |  |
| ReAgg | 0.423 | 0.047 | 8.966 | 0.000 | 0.331 | 0.516 |


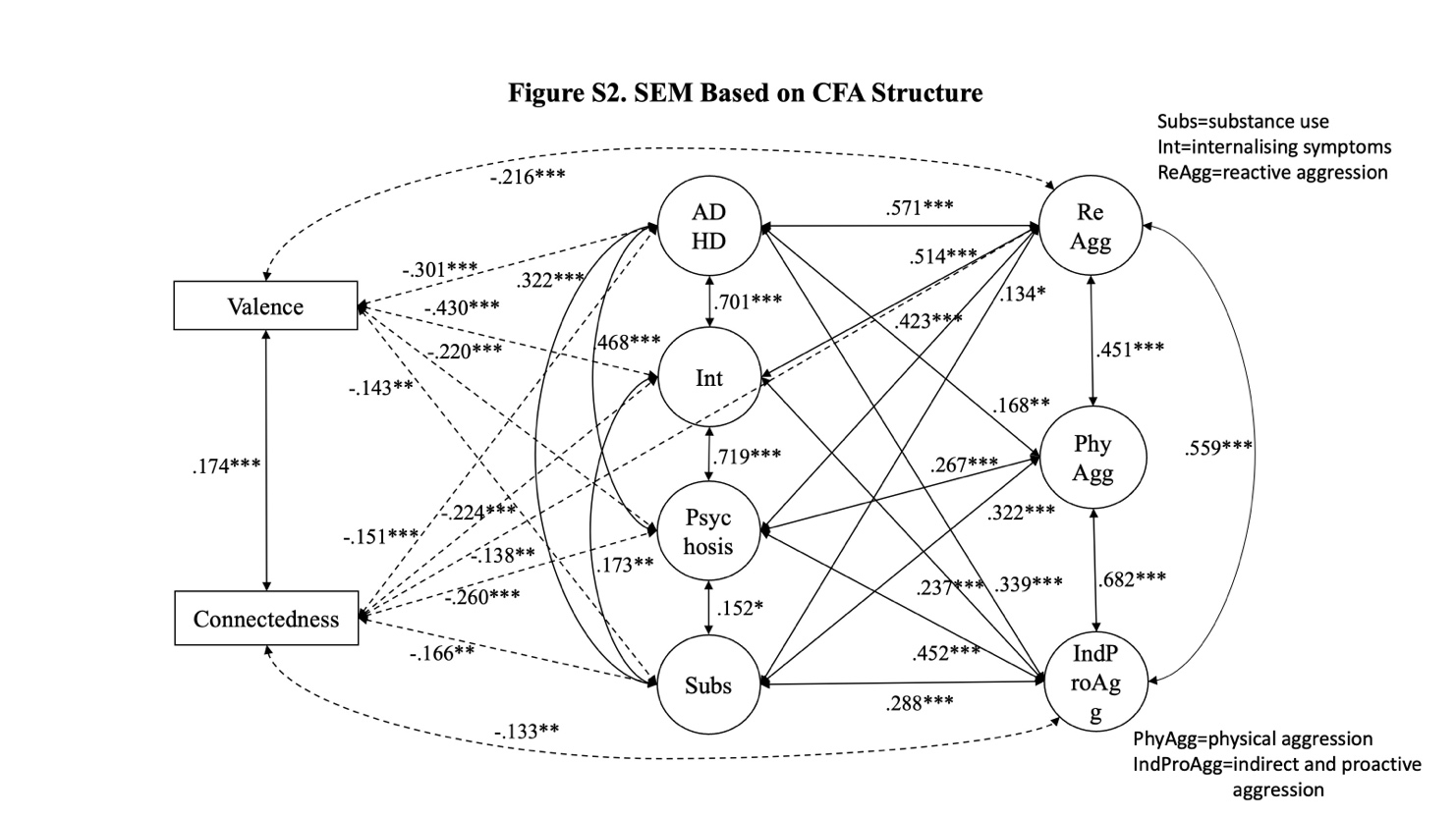


| **Table S9. Substance use SEM with 95%CL (N=587)** | | | | | | |
| --- | --- | --- | --- | --- | --- | --- |
| Latent Variables: | est.std | Std.Err | z-value | P(>\|z\|) | ci.lower | ci.upper |
| Subs =~ |  |  |  |  |  |  |
| Subs1 | 0.715 | 0.039 | 18.146 | 0.000 | 0.637 | 0.792 |
| Subs2 | 0.892 | 0.020 | 44.601 | 0.000 | 0.852 | 0.931 |
| Subs3 | 0.863 | 0.025 | 34.876 | 0.000 | 0.814 | 0.911 |
| Subs4 | 0.872 | 0.026 | 33.864 | 0.000 | 0.822 | 0.923 |
| Subs7 | 0.770 | 0.036 | 21.423 | 0.000 | 0.700 | 0.841 |
| Subs5 | 0.922 | 0.023 | 40.965 | 0.000 | 0.878 | 0.966 |
| Subs6 | 0.565 | 0.049 | 11.454 | 0.000 | 0.468 | 0.662 |
|  |  |  |  |  |  |  |
| Covariances: | est.std | Std.Err | z-value | P(>\|z\|) | ci.lower | ci.upper |
| Subs ~~ |  |  |  |  |  |  |
| Self-valence | -0.144 | 0.052 | -2.773 | 0.006 | -0.246 | -0.042 |
| Self-connectedness | -0.164 | 0.050 | -3.259 | 0.001 | -0.263 | -0.065 |
| .Self-connectedness ~~  .self-valence | 0.174 | 0.040 | 4.375 | 0.000 | 0.096 | 0.252 |

| **Table S10. ADHD SEM with 95%CL (N=587)** | | | | | | |
| --- | --- | --- | --- | --- | --- | --- |
| Latent Variables: | est.std | Std.Err | z-value | P(>\|z\|) | ci.lower | ci.upper |
| ADHD =~ |  |  |  |  |  |  |
| SBQ15 | 0.777 | 0.017 | 45.764 | 0.000 | 0.744 | 0.810 |
| SBQ16 | 0.834 | 0.015 | 55.047 | 0.000 | 0.804 | 0.864 |
| SBQ74 | 0.683 | 0.023 | 29.054 | 0.000 | 0.637 | 0.729 |
| SBQ12 | 0.673 | 0.023 | 28.960 | 0.000 | 0.627 | 0.718 |
| SBQ17 | 0.773 | 0.020 | 38.697 | 0.000 | 0.734 | 0.812 |
| SBQ13 | 0.740 | 0.021 | 34.817 | 0.000 | 0.698 | 0.781 |
| SBQ14 | 0.717 | 0.021 | 33.746 | 0.000 | 0.675 | 0.759 |
| SBQ10 | 0.567 | 0.029 | 19.523 | 0.000 | 0.510 | 0.624 |
| SBQ73 | 0.610 | 0.026 | 23.125 | 0.000 | 0.558 | 0.662 |
|  |  |  |  |  |  |  |
| Covariances: | est.std | Std.Err | z-value | P(>\|z\|) | ci.lower | ci.upper |
| ADHD ~~ |  |  |  |  |  |  |
| Self-valence | -0.301 | 0.041 | -7.385 | 0.000 | -0.380 | -0.221 |
| Self-connectedness | -0.150 | 0.041 | -3.617 | 0.000 | -0.231 | -0.069 |
| .Self-connectedness ~~  .self-valence | 0.174 | 0.040 | 4.375 | 0.000 | 0.096 | 0.252 |

| **Table S11. Internalising problems SEM with 95%CL (N=587)** | | | | | | |
| --- | --- | --- | --- | --- | --- | --- |
| Latent Variables: | est.std | Std.Err | z-value | P(>\|z\|) | ci.lower | ci.upper |
| Int =~ |  |  |  |  |  |  |
| SBQ08 | 0.811 | 0.018 | 45.965 | 0.000 | 0.776 | 0.845 |
| SBQ64 | 0.811 | 0.017 | 48.978 | 0.000 | 0.778 | 0.843 |
| SBQ05 | 0.717 | 0.023 | 31.438 | 0.000 | 0.672 | 0.762 |
| SBQ85 | 0.848 | 0.014 | 58.738 | 0.000 | 0.820 | 0.876 |
| SBQ83 | 0.860 | 0.015 | 56.156 | 0.000 | 0.830 | 0.890 |
| SBQ84 | 0.859 | 0.017 | 51.379 | 0.000 | 0.826 | 0.892 |
| SBQ03 | 0.740 | 0.024 | 30.874 | 0.000 | 0.693 | 0.787 |
| SBQ04 | 0.797 | 0.017 | 47.169 | 0.000 | 0.764 | 0.831 |
| SBQ81 | 0.740 | 0.021 | 35.167 | 0.000 | 0.699 | 0.782 |
| SBQ63 | 0.479 | 0.032 | 15.047 | 0.000 | 0.417 | 0.541 |
| SBQ82 | 0.608 | 0.028 | 21.342 | 0.000 | 0.552 | 0.664 |
| SBQ01 | 0.658 | 0.025 | 26.780 | 0.000 | 0.610 | 0.706 |
| SBQ62 | 0.582 | 0.029 | 19.917 | 0.000 | 0.525 | 0.639 |
| SBQ79 | 0.638 | 0.036 | 17.566 | 0.000 | 0.567 | 0.709 |
|  |  |  |  |  |  |  |
| Covariances: | est.std | Std.Err | z-value | P(>\|z\|) | ci.lower | ci.upper |
| Int ~~ |  |  |  |  |  |  |
| Self-valence | -0.431 | 0.037 | -11.534 | 0.000 | -0.504 | -0.357 |
| Self-connectedness | -0.229 | 0.041 | -5.648 | 0.000 | -0.309 | -0.150 |
| .Self-connectedness ~~  .self-valence | 0.174 | 0.040 | 4.374 | 0.000 | 0.096 | 0.252 |

| **Table S12. Physical aggression SEM with 95%CL (N=587)** | | | | | | |
| --- | --- | --- | --- | --- | --- | --- |
| Latent Variables: | est.std | Std.Err | z-value | P(>\|z\|) | ci.lower | ci.upper |
| PhyAgg =~ |  |  |  |  |  |  |
| SBQ34 | 0.925 | 0.021 | 43.652 | 0.000 | 0.884 | 0.967 |
| SBQ35 | 0.908 | 0.023 | 39.868 | 0.000 | 0.864 | 0.953 |
| SBQ33 | 0.890 | 0.025 | 35.767 | 0.000 | 0.841 | 0.939 |
| SBQ54 | 0.734 | 0.031 | 23.442 | 0.000 | 0.673 | 0.796 |
| SBQ72 | 0.603 | 0.040 | 15.030 | 0.000 | 0.525 | 0.682 |
| SBQ37 | 0.806 | 0.040 | 20.062 | 0.000 | 0.727 | 0.884 |
|  |  |  |  |  |  |  |
| Covariances: | est.std | Std.Err | z-value | P(>\|z\|) | ci.lower | ci.upper |
| PhyAgg ~~ |  |  |  |  |  |  |
| Self-valence | -0.080 | 0.052 | -1.532 | 0.126 | -0.182 | 0.022 |
| Self-connectedness | -0.011 | 0.048 | -0.219 | 0.827 | -0.105 | 0.084 |
| .Self-connectedness ~~  .self-valence | 0.174 | 0.040 | 4.375 | 0.000 | 0.096 | 0.252 |

| **Table S13. Indirect and proactive aggression SEM with 95%CL (N=587)** | | | | | | |
| --- | --- | --- | --- | --- | --- | --- |
| Latent Variables: | est.std | Std.Err | z-value | P(>\|z\|) | ci.lower | ci.upper |
| IndProAgg =~ |  |  |  |  |  |  |
| SBQ21 | 0.607 | 0.036 | 16.788 | 0.000 | 0.536 | 0.678 |
| SBQ52 | 0.752 | 0.033 | 22.497 | 0.000 | 0.686 | 0.817 |
| SBQ19 | 0.716 | 0.036 | 20.032 | 0.000 | 0.646 | 0.787 |
| SBQ22 | 0.748 | 0.039 | 19.379 | 0.000 | 0.673 | 0.824 |
| SBQ23 | 0.633 | 0.053 | 11.905 | 0.000 | 0.529 | 0.737 |
| SBQ61 | 0.788 | 0.036 | 21.996 | 0.000 | 0.718 | 0.858 |
| SBQ51 | 0.558 | 0.042 | 13.171 | 0.000 | 0.475 | 0.641 |
| SBQ37 | 0.747 | 0.049 | 15.149 | 0.000 | 0.650 | 0.844 |
|  |  |  |  |  |  |  |
| Covariances: | est.std | Std.Err | z-value | P(>\|z\|) | ci.lower | ci.upper |
| IndProAgg ~~ |  |  |  |  |  |  |
| Self-valence | -0.078 | 0.051 | -1.540 | 0.124 | -0.178 | 0.021 |
| Self-connectedness | -0.120 | 0.049 | -2.439 | 0.015 | -0.216 | -0.024 |
| .Self-connectedness ~~  .self-valence | 0.174 | 0.040 | 4.375 | 0.000 | 0.096 | 0.252 |

| **Table S14. Psychosis SEM with 95%CL (N=587)** | | | | | | |
| --- | --- | --- | --- | --- | --- | --- |
| Latent Variables: | est.std | Std.Err | z-value | P(>\|z\|) | ci.lower | ci.upper |
| Psychosis =~ |  |  |  |  |  |  |
| SBQ76 | 0.799 | 0.044 | 18.010 | 0.000 | 0.712 | 0.886 |
| SBQ79 | 0.733 | 0.041 | 18.091 | 0.000 | 0.653 | 0.812 |
| SBQ71 | 0.682 | 0.060 | 11.405 | 0.000 | 0.565 | 0.799 |
| SBQ80 | 0.722 | 0.040 | 18.106 | 0.000 | 0.644 | 0.800 |
| SBQ77 | 0.711 | 0.045 | 15.710 | 0.000 | 0.622 | 0.800 |
| SBQ75 | 0.637 | 0.059 | 10.867 | 0.000 | 0.522 | 0.752 |
|  |  |  |  |  |  |  |
| Covariances: | est.std | Std.Err | z-value | P(>\|z\|) | ci.lower | ci.upper |
| Psychosis ~~ |  |  |  |  |  |  |
| Self-valence | -0.222 | 0.054 | -4.088 | 0.000 | -0.329 | -0.116 |
| Self-connectedness | -0.264 | 0.050 | -5.256 | 0.000 | -0.362 | -0.165 |
| .Self-connectedness ~~  .self-valence | 0.174 | 0.040 | 4.375 | 0.000 | 0.096 | 0.252 |

| **Table S15. Reactive aggression SEM with 95%CL (N=587)** | | | | | | |
| --- | --- | --- | --- | --- | --- | --- |
| Latent Variables: | est.std | Std.Err | z-value | P(>\|z\|) | ci.lower | ci.upper |
| ReAgg =~ |  |  |  |  |  |  |
| SBQ53 | 0.777 | 0.029 | 27.012 | 0.000 | 0.720 | 0.833 |
| SBQ55 | 0.765 | 0.035 | 21.883 | 0.000 | 0.697 | 0.834 |
| SBQ66 | 0.683 | 0.031 | 22.029 | 0.000 | 0.622 | 0.743 |
| SBQ01 | 0.347 | 0.044 | 7.926 | 0.000 | 0.261 | 0.433 |
| SBQ72 | 0.708 | 0.032 | 22.339 | 0.000 | 0.645 | 0.770 |
|  |  |  |  |  |  |  |
| Covariances: | est.std | Std.Err | z-value | P(>\|z\|) | ci.lower | ci.upper |
| ReAgg ~~ |  |  |  |  |  |  |
| Self-valence | -0.216 | 0.044 | -4.964 | 0.000 | -0.301 | -0.131 |
| Self-connectedness | -0.152 | 0.046 | -3.309 | 0.001 | -0.242 | -0.062 |
| .Self-connectedness ~~  .self-valence | 0.174 | 0.040 | 4.375 | 0.000 | 0.096 | 0.252 |

| **Table S16. Preregistered and Data-Dependent Analytic Steps** | | | |
| --- | --- | --- | --- |
| **Analytic Component** | **Preregistered** | **Implementation in Present Study** | **Notes / Deviations** |
| Sample selection (Wave 8; age ≈ 20) | Yes | Restricted to Wave 8 | Future-oriented measures available only at Wave 8 |
| Measured variables (SBQ, psychosis-like symptoms, substance use, future orientation) | Yes | Analysed as specified | Future orientation operationalised using available indicators |
| Exploratory factor analysis (EFA) | Not explicitly specified | Conducted in calibration subsample | Measurement validation prior to bifactor modelling |
| Confirmatory factor analysis (CFA) | Not explicitly specified | Conducted in validation subsample | Measurement validation prior to bifactor modelling |
| Symmetrical bifactor modelling | Yes | Estimated and evaluated | Core analytic plan |
| Model selection based on fit and interpretability | Yes | Global fit indices and item-level diagnostics applied | Criteria consistent with preregistered bifactor plan |
| Evaluation of S-1 bifactor specification | Not explicitly specified | Evaluated within broader bifactor framework | Introduced following model diagnostics |
| Structural equation modelling (SEM) associations with future orientation components | Yes | Conducted using selected optimal measurement model | No change in substantive hypotheses |
| Robustness analyses | Partially specified | Additional SEMs across alternative frameworks | Expanded for transparency |
| *Note: The preregistration specified the use of bifactor modelling to examine shared versus symptom-domain-specific variance and to test associations between future orientation and both general and specific factors. The preregistration was completed prior to data access and described future orientation at a conceptual level. Exploratory and confirmatory factor analyses were conducted to establish and validate the measurement structure prior to bifactor modelling. The S-1 bifactor specification was not explicitly named in the preregistration. It was evaluated within the broader preregistered bifactor modelling framework and selected based on prespecified model comparison criteria (fit and interpretability). No substantive changes were made to the core hypotheses.* | | | |
